# The effect of deep magnetic stimulation on the cardiac-brain axis post-sleep deprivation: a pilot study

**DOI:** 10.3389/fnins.2024.1464299

**Published:** 2025-01-10

**Authors:** Qiongfang Cao, Peng Zhang, Fangfang Liu, Mengyan Jin, Yuhan Wang, Hanrui Zeng, Xiechuan Weng, Fan Xu

**Affiliations:** ^1^Department of Evidence-Based Medicine and Social Medicine, School of Public Health, Chengdu Medical College, Chengdu, Sichuan, China; ^2^Art College of Southwest Minzu University, Chengdu, Sichuan, China; ^3^Department of Internal Medicine, West China Hospital of Sichuan University, Chengdu, China; ^4^Department of Radiology, Sichuan Taikang Hospital, Chengdu, Sichuan, China; ^5^Department of Neuroscience, Beijing Institute of Basic Medical Sciences, Beijing, China

**Keywords:** heart sound, deep magnetic stimulation (DMS), sleep deprivation, functional magnetic resonance imaging (fMRI), cardiac-brain axis

## Abstract

**Introduction:**

Sleep deprivation (SD) significantly disrupts the homeostasis of the cardiac-brain axis, yet the neuromodulation effects of deep magnetic stimulation (DMS), a non-invasive and safe method, remain poorly understood.

**Methods:**

Sixty healthy adult males were recruited for a 36-h SD study, they were assigned to the DMS group or the control group according to their individual willing. All individuals underwent heart sound measurements and functional magnetic resonance imaging scans at the experiment’s onset and terminal points. During the recovery sleep phase, DMS was applied twice for 30 min before sleep onset and upon awakening to the individuals in the DMS group. Two-factor analysis was used to disclose the changes in two status and intervention effect in groups, along with Spearman rank correlation analysis to assess the correlation between brain activity and heart activity, the linear regression analysis was performed to explore the effect of DMS on brain regions to regulated the heart activity. Additionally, bootstrapping analysis was employed to verify the mediation effect.

**Results:**

The results indicated that the DMS group cardiac cycle duration was 0.81 ± 0.04 s, CON group was 0.80 ± 0.03 s, DMS presented a prolong effect (*F* = 0.32, *p* = 0.02), and all heart frequency and intensity indexes value were lower than CON group (*p* < 0.01). Two-factor analysis demonstrated the significant differences in the left insula and orbitofrontal inferior gyrus, which DC_Weight (0.25) value were lower 0.50 (*p* < 0.01), 0.42 (*p* < 0.01) after DMS. Furthermore, the correlation analysis confirmed that the negative association between the left orbital inferior frontal and left insula with the heart sound index (*p* < 0.05), such as Δ left orbital inferior frontal were negatively correlated with Δ Systolic_intensity (rho = −0.33, *p* < 0.05), Δ Diastolic_intensity (rho = −0.41, *p* < 0.05), Δ S1_intensity (rho = −0.36, *p* < 0.05), and Δ S2_intensity (rho = −0.43, *p* < 0.05). Δ Left insula was negatively correlated with Δ Diastolic_intensity (rho = −0.36, *p* < 0.05), Δ S1_intensity (rho = −0.33, *p* < 0.05), and Δ S2_intensity (rho = −0.36, *p* < 0.05). Mediated effect analysis showed that DMS affected S2_intensity by intervening in brain regions.

**Conclusion:**

These findings suggest a causal effect on the cardiac-brain axis following 36 h of SD. The non-invasive intervention of DMS effectively regulates both brain and heart functions after SD, promoting homeostatic balance. The DMS can affect the cardiac-brain axis, offering a means to restore balance following extended periods of SD.

## Introduction

Sleep Deprivation (SD) is a widespread public health challenge, impacting various occupations and has become a prevalent issue in contemporary society. Notably, it contributes to the development of insomnia, a condition associated with a twofold increase in the risk of depression compared to individuals without sleep disturbances, constituting a significant risk factor for depression and common clinical symptoms (Staner, 2010; Baglioni et al., 2011; Chung et al., 2015; Ribeiro et al., 2018; Yaremchuk, 2018; Dare et al., 2019; Van Someren, 2021). Prolonged insomnia elevates the risk of depression and anxiety disorders (Wittchen et al., 2011; Maier et al., 2021; Lv et al., 2023), affecting approximately 7% of EU adults (Roth et al., 2011), 9%–20% of US adults (Singareddy et al., 2012), and 7%–10% of UK adults, with a prevalence of 37% (Pawlyk et al., 2008). The global prevalence of insomnia reflects its epidemic nature (Baldwin and Daugherty, 2004), detrimentally impacting psychological wellbeing (Palagini et al., 2013)and cardiac metabolic health (Daley et al., 2009), thereby imposing a substantial economic burden (Palagini et al., 2013).

While SD remains inevitable in certain occupations, the extended or irregular working hours exacerbate mental health issues for workers (Sato et al., 2020). Occupations such as nursing, long-distance driving, and intercontinental piloting may lead to reduced prefrontal cortex activity after irregular work and rest, resulting in cognitive function decline, particularly when night shifts disrupt normal circadian physiological functions (Zare Khormizi et al., 2019; Khan et al., 2020). Numerous studies affirm that sleep disorders, or simply insufficient sleep patterns, are strongly linked to cardiovascular disease complications, including high blood pressure, obesity, diabetes, and dyslipidemia (Karlsson et al., 2001; Knutson et al., 2007; Cappuccio et al., 2011). Short sleep duration is notably associated with an increased risk of developing or succumbing to cardiovascular disease (Nagai et al., 2010). Epidemiological data and prior SD experiments emphasize that SD can adversely impact metabolism, the cardiovascular system, and immunity (Dinges et al., 1994). For instance, increased white blood cell (Faraut et al., 2011) and neutrophil counts (GBD 2016 DALYs and HALE Collaborators, 2017) emerge as independent risk factors for cardiovascular death following SD or severe sleep restriction. Moreover, insufficient sleep accelerates the early deterioration of blood vessel structure and function, leading to increased arterial stiffness, thereby influencing responses to cardiovascular stimuli such as pain, stress, or mood. More critically, it disrupts circadian rhythms and autonomic nervous balance, influencing cardiac output rhythms and elevating blood pressure variability (Gangwisch, 2014).

Short periods of SD can induce sympathetic overactivity, leading to increased blood pressure, heart rate, and heightened production of stress hormones such as cortisol, norepinephrine, and thyroid hormone. Elevated stress hormone levels and enhanced cardiac contractility contribute to the activation of the sympathetic nervous system, influencing hormone secretion and blood pressure regulation. Acute short-term SD has been shown to impair cardiac artery endothelial function within 24 h (Kuetting et al., 2019). Physiological connections between the heart and brain, termed the heart-brain axis, are bidirectional, occur through a complex network of autonomic nerves/hormones and cytokines. It plays important roles in different medical conditions (Hu et al., 2023). Our previous study validated that SD affects the function of the brain and heart, and also closely linked in the heart-brain axis (Manea et al., 2015; Cao et al., 2024). From the physiology perspective, blood fills the ventricle during ventricular diastole in each cardiac cycle when the heart at its most relaxed state, then left ventricle ejected the blood into the circulatory system during ventricular systole (when the ventricles contract maximally). During peak systolic periods, blood pressure reaches its highest point, triggering stretch-response baroreceptors located in the carotid sinus and aortic arch. These receptors may activated in response to the intensity and timing of heart contractions (Craig, 2009; Critchley and Harrison, 2013; Duschek et al., 2013), and the baroreceptor signal is transmitted through the vagus and glossopharyngeal nerves to the nucleus of the brain stem’s solitary-tract (NTS), which regulates blood pressure and heart rate through baroreflex (Engelen et al., 2023). Then, it progresses to subcortical structures such as the thalamus, amygdala, and hypothalamus, and subsequently to the central cortical region. This process contains the insular cortex, cingulate cortex, and the primary and secondary somatosensory cortex (Park and Tallon-Baudry, 2014; Pramme et al., 2014; Pramme et al., 2016; Grund et al., 2022). Moreover, another study validated that the involuntary motor evoked potentials triggered by transcranial magnetic stimulation (TMS) were larger during systole, suggesting a motion-promoting effect when baroreceptor activity increased (Al et al., 2023). Therefore, from the working principles, deep magnetic stimulation (DMS) interventions may be also influenced along the cardio-brain axis.

Deep magnetic stimulation is a non-invasive therapeutic approach that delivers continuous electromagnetic stimulation to specific brain regions, modulating abnormal neural circuits and providing therapeutic benefits for various diseases (Harmsen et al., 2020). DMS generates gamma burst oscillation, producing magnetic field output pulses with higher frequency, lower intensity, and a broader range compared to other magnetic stimuli. Our previous study demonstrated the effective stimulation of brain structures, particularly the hippocampus, in rodent models, resulting in improved learning and memory (Zhen et al., 2017). Despite these advancements, the mechanisms and effects of DMS on the cardiac-brain axis following SD are poorly understood. In this study, 60 healthy male participants were recruited for a 36-h SD, to further validate the treatment mechanism of DMS on recovery.

## Materials and methods

### Ethic statement

The study was approved by the Medical and Health Research Ethics Committee of the Biological and Medical Ethics Committee of Beihang University, Beijing, China, under Approval No. BM20180040, and it was conducted in accordance with the Declaration of Helsinki.

### Individuals

1. Recruitment process: from August 2021 to August 2022, we recruited students from Chengdu Medical College and University of Electronic Science and Technology of China. A total of 402 healthy college students with good work and rest, aged 18–28, were enrolled and passed the preliminary examination. A total of 192 qualified individuals were screened according to the exclusion criteria.
2. The inclusion criteria were as follows: (a) aged 18–28 years; (b) right-handed; (c) maintaining a regular sleep schedule (approximately 8 h of sleep per night); (d) no history of cardiovascular diseases, respiratory diseases, nervous system diseases, psychiatric disorders, or sleep disorders, established by interviews, questionnaires, and physical examinations; and (e) non-smokers and non-alcoholics. Participants were informed to maintain regular sleep schedules and keep a sleep diary 1 week before the experiment, such as when go to bed, when wake up, and the amount duration of sleep each day. Consuming caffeine, tobacco, alcohol, or tea, and taking medicine were prohibited within 1 week before and throughout the experiment.
3. The exclusion criteria were as follows: BDI ≥ 13 (Beck et al., 1961) and BAI 45 (Beck et al., 1988) were applied as exclusion criteria, based on which participants with depressive disorder and anxiety were excluded.
4. Grouping: in this study, 30 participants were assigned to the DMS group and 30 to the CON group, based on their willingness to participate and their fulfillment of the inclusion and exclusion criteria, until the target enrollment was achieved.

### Experimental flow

**FIGURE 1:**
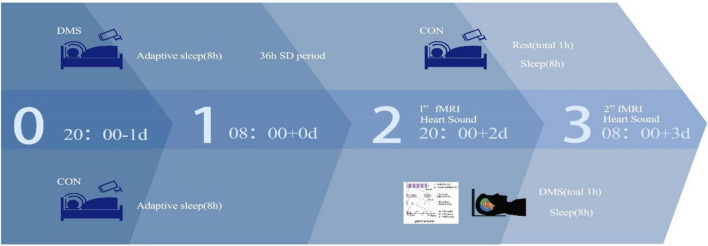
Experimental flow.

The SD protocol involved the individuals arriving 1 day in advance to acclimate to the new surroundings from 22:00 on the joining group’s day to 08:00 on the first day. The SD experiment began at 08:00 on day 1 and ended at 20:00 on day 2. Throughout this period, experienced staff ensured that each individual remained awake. Following SD, functional magnetic resonance imaging (fMRI) and heart sound data were collected from both groups. After the end of SD on day 2, the CON group resumed sleep from 22:30 to 08:00 on day 2. Simultaneously, the DMS group received DMS stimulation for 30 min from 22:00 to 22:30 and initiated recovery sleep at 22:30. A camera system monitored the entire sleep period to guarantee both groups received a minimum of 8 h of restorative sleep. Post 08:00 on day 3, the DMS group underwent another 30-min DMS session, and the second round of fMRI and heart sound data were collected for both groups. Throughout the experiment, individuals remained in the sleep lab, refraining from strenuous activities or consuming stimulant-containing substances or beverages. Staff members continuously monitored individuals to ensure wakefulness during SD. Refer to Figure 1 for a detailed workflow of the experiment.

### Primary outcome

As this study was an exploratory pilot study, the primary outcome endpoint of this study was the effect of DMS on heart sound indexes; secondary endpoints included: (1) fMRI data indicators ALFF, ReHo, DC; (2) heart sound cardiac cycle frequency, intensity; (3) systolic cycle, diastolic cycle, s1 cycle and s2 cycle duration, frequency, and intensity; and (4) the change data (Δ) in fMRI and heart sound data between before and after intervention.

### Deep magnetic stimulation

The DMS equipment, designed and manufactured by Beijing Aldans Biotech Co., Ltd., took the form of a laptop with two sides containing a 360 mm-diameter coil. This apparatus was linked to a magnetic field generator, generating a time-varying magnetic field. Each 2-s output consisted of rhythmic trains with intervals of 27, 25, 23, 21, or 19 ms, forming intermittent gamma burst stimulation at a frequency of 30–40 Hz. Each train comprised six pulses with a width of 130 ms and a frequency of 1,000 Hz. These 2-s runs were interspersed with an 8-s resting interval. Additionally, the shape of the magnetic fields changed every 4 min (alternating between linear gradient and approximate distribution), with the rhythm gradually increasing every 8 min (30, 32.25, 34.5, 37, and 40 Hz). For further details, refer to our previous study (Zhen et al., 2017).

### Heart sound

Data were collected using an ETZ-1A(C) electronic stethoscope (Exagiga Electric Co., LTD., China). The following steps were undertaken: (1) the stethoscope was disinfected before use, and the probe was placed on the palm for 1–2 min; (2) in a quiet environment, individuals assumed a supine, lateral, or sitting position with their chest exposed; (3) the auscultation head was inserted into the headphone jack of the mobile phone (some models required a converter); (4) the auscultation heads on the five valve areas were sequentially pressed: the mitral valve area (first auscultation area), the aortic valve area (second auscultation area), the main A valve (third auscultation area), the pulmonary valve area (fourth auscultation area), and the tricuspid valve area (fifth auscultation area). The mobile phone recorder was then activated for recording; (5) finally, the test individuals saved the audio, named after the heart sound record as the date-student number-auscultation area. Data for each individual were collected every 2 h, with 60 s for each auscultation area. Audacity software (version 1.3.3) was employed to segment heart sounds into S1, S2, systolic, and diastolic periods, extract acoustic indexes for each segment, and obtain duration, intensity, and frequency indexes for each segment through principal component analysis. The duration, frequency and intensity of the cardiac cycle, systole, diastole, s1, and s2 were collected. Further details about the heart sound analysis process can be found in our previous studies (Cao et al., 2024) and Supplementary File 1.

### fMRI data

The preprocessing of data was conducted using MATLAB 2020a. Micron was employed to convert the raw DICOM data format. Dpabi2.3 was utilized for preprocessing the Nii data, involving removal of the initial 10 time points, slice timing correction (Parker and Razlighi, 2019), inspection and correction of head movement, segmentation registration (DARTEL), and spatial standardization (333). The average head movement for each individual was calculated post head movement correction. Individuals exceeding a head movement translation of >2 mm and/or head movement rotation of >2° were excluded based on the rotated head movement diagram (no individuals’ data exceeded the specified range) (Strother, 2006). Subsequently, the Restplus package was used to calculate the whole-brain DC map of the preprocessed data. This involved removing the first-order linear trend, regressing covariates, low-frequency filtering (0.01–0.08 Hz), and applying 6 × 6 × 6 Gaussian smoothing (Wang et al., 2021). All results were subjected to statistical analysis after z-standard transformation. Further details about the fMRI FC data acquisition and analysis process can be found in Supplementary File 4.

### Statistical analysis

All data were analyzed using Stata 18.0. Continuous variables with a normal distribution were expressed as mean ± standard deviation, while those not conforming to a normal distribution were presented as median (Q1, Q3). Two-way ANOVA was applied for variables with normal distribution, and non-parametric rank sum tests were utilized for variables not satisfying normality. If no interaction effect was observed, main effect analysis was performed; otherwise, a simple effect analysis was conducted. Pearson correlation analysis was used when the variable data were continuous and normality distributed. Otherwise, Spearman rank correlation analysis was used to explore the relationship between groups, heart sounds, and DMS indicators. Bonferroni correction was applied as the method for multiple comparisons correction. The mediating effect was assessed with stepwise linear regression analysis and deviation-corrected percentile bootstrapping. A significance level of *p* < 0.05 was considered statistically significant.

## Results

### Demographic

Sixty healthy male volunteers, with an average age of 21.96 ± 1.76 years, were recruited for the study (Table 1). All participants were right-handed and devoid of mental illness, cardiovascular diseases, neurological disorders, head trauma, sleep disorders, seizures, or smoking habits, BDI < 13, BAI < 45, and no depressive disorder or anxiety.

**TABLE 1:** Basic information.

Variable	All (*n* = 60)	DMS group (*n* = 30)	CON group (*n* = 30)	*t/Z*	*p*
Age (years)	21.96 ± 1.76	21.83 ± 1.78	22.10 ± 1.94	−0.56	0.58
BMI (Kg/m^2^)	21.29 ± 2.23	22.57 ± 2.92	20.79 ± 2.49	2.53	0.01

BMI, body mass index; CON, control; DMS, deep magnetic stimulation.

### Analysis of the cardiac index – heart sound

#### Changes in the heart sound between SD 36 h and RS in groups

At SD 36 h point, the parameters of heart sound, including s1_duration, systolic_intensity, diastolic_intensity, s1_intensity, and s2_intensity the between two groups were significant different. While the paired *t*-test results showed that only s2_duration was significant different in the DMS group between before and after intervention, meanwhile there was no significant difference in the heart sound data of CON group between pre and post sleep. Then, the change data shows that only Δ s2_duration was significant different between two groups, Δ s2_duration in DMS group presented significant more than CON group (details see Table 1 in Supplementary File 2).

#### Bivariate analysis of the cardiac index – heart sound

**FIGURE 2:**
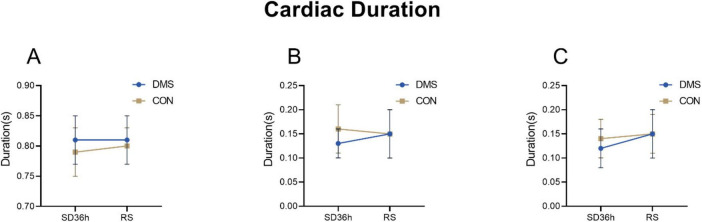
Cardiac duration at different time points in the DMS group and CON group. **(A)** *Cardiac cycle duration*, DMS group’s cardiac cycle duration prolong than CON group (*F* = 0.32, *p* = 0.02). **(B)** *S1_duration*, S1_duration’ difference was not statistically significant (*F* = 2.97, *p* = 0.09). **(C)** *S2_duration*, S2_duration’ difference was not statistically significant (*F* = 0.99, *p* = 0.32). The error bars indicate the standard deviation, the larger the error bars, the greater the variability or uncertainty in the data. The *Y*-axis unit is second (s).

**FIGURE 3:**
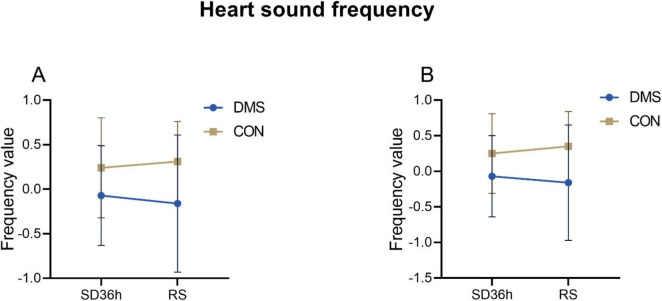
Heart sound frequency at different time points in the DMS group and CON group. **(A)** *Systolic_frequency*, DMS group’s systolic_frequency lower than CON group (*F* = 9.66, *p* < 0.01). **(B)** *Diastolic_frequency*, DMS group’s diastolic_frequency lower than CON group (*F* = 10.62, *p* < 0.01). The error bars indicate the standard deviation, the larger the error bars, the greater the variability or uncertainty in the data. Those indexes were PCA dimension reduction indexes with no units.

**FIGURE 4:**
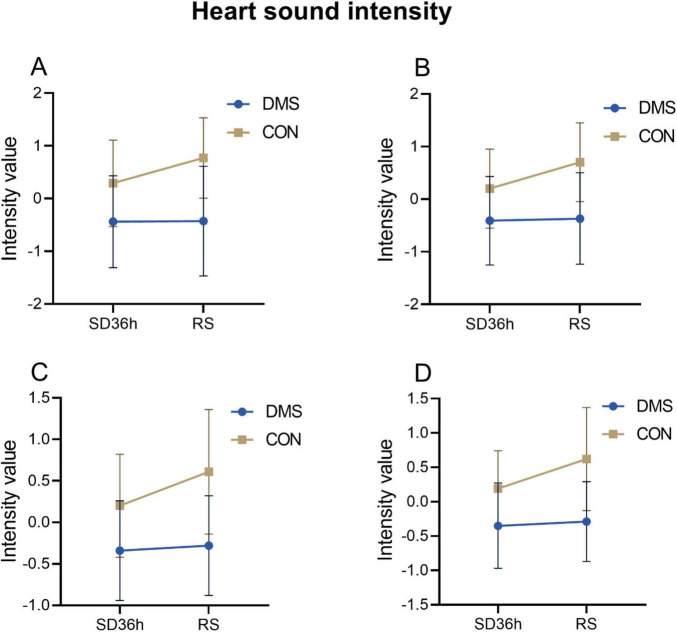
Heart sound intensity at different time points in the DMS group and CON group. **(A)** *S1_ intensity*, DMS group’s S1_ intensity lower than CON group (*F* = 29.06, *p* < 0.01). **(B)** *S2_ intensity*, DMS group’s S2_intensity lower than CON group (*F* = 30.81, *p* < 0.01). **(C)** *Systolic_intensity*, DMS group’s systolic_intensity lower than CON group (*F* = 27.73, *p* < 0.01). **(D)** *Diastolic_intensity*, DMS group’s diastolic_intensity lower than CON group (*F* = 25.31, *p* < 0.01). The error bars indicate the standard deviation, the larger the error bars, the greater the variability or uncertainty in the data. Those indexes were PCA dimension reduction indexes with no units.

The two-factor analysis results revealed a significant group effect on cardiac cycle duration [the process that the cardiovascular system undergoes from the start of one heartbeat to the start of the next (Ashley and Niebauer, 2004)], systolic frequency, diastolic frequency, systolic intensity, diastolic intensity, S1 intensity, and S2 intensity (details see Supplementary File 1). No interaction effect was observed (*p* > 0.05). Main effect analysis indicated that the DMS group presented a significant longer cardiac cycle duration (*F* = 0.32, *p* = 0.02), lower heart systolic frequency (*F* = 9.66, *p* < 0.01) and diastolic frequency (*F* = 10.62, *p* < 0.01), reduced heart systolic intensity (*F* = 29.06, *p* < 0.01), diastolic intensity (*F* = 30.81, *p* < 0.01), s1 intensity (*F* = 27.73, *p* < 0.01), and s2 intensity (*F* = 25.31, *p* < 0.01) compared to the CON group (refers to Figures 2–4, Tables 2, 3 in Supplementary File 2). Similar results were observed when analyzing heart sound data in other auscultation areas, as detailed in Supplementary File 3.

**TABLE 2:** Simple effect analysis of the DC_Weight value of the left insula at the fixed group level.

Group	Session 1	Session 2	Difference	SE	*p*
CON	SD 36 h	RS	−0.20	0.12	0.28
DMS	SD 36 h	RS	0.50	0.12	<0.01

The significance level of the mean difference is 0.05. Multiple comparison regulation: Bonferroni method.

**TABLE 3:** Simple effect analysis of the DC_Binary value of the left orbital inferior frontal gyrus at the fixed group level.

Group	Session 1	Session 2	Difference	SE	*p*
CON	SD 36 h	RS	−0.21	0.116	0.24
DMS	SD 36 h	RS	0.42	0.116	<0.01

The significance level of the mean difference is 0.05. Multiple comparison by Bonferroni method.

### Bivariate analysis of brain indicators-fMRI

**FIGURE 5:**
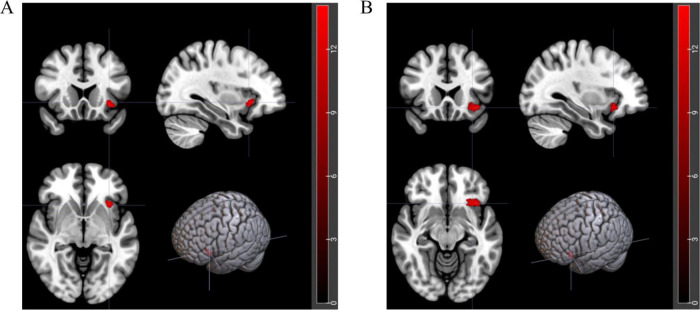
Brain regions with interactive effects after two-factor analysis. **(A)** Red area represents left orbital inferior frontal gyrus (*t* = 23.01, *p* < 0.01). **(B)** Red area represents left insula (*t* = 25.75, *p* < 0.01).

Bivariate analysis was conducted on each MRI index, revealing differential clusters only in DC_Weight 0.25 and DC_Binary 0.25, which included the left orbital inferior frontal gyrus (*t* = 23.01, *p* < 0.01) and left insula (*t* = 25.75, *p* < 0.01). The DC values of these regions were extracted for further bivariate analysis. A two-factor analysis of the DC_Weight (0.25) value of the left insula indicated interactive effects between status and group on this brain region. Simple effect analysis revealed that, when the group was fixed as DMS, the DC value after recovery sleep (0.50) was significantly lower than that after SD. Similarly, a two-factor analysis of the DC_Binary (0.25) value of the left orbital inferior frontal gyrus showed interactive effects between status and group on this brain region. The simple effect analysis demonstrated that, when the fixed group was DMS, the DC value after recovery sleep (0.42) was significantly lower than that after SD (details see Figure 5, Tables 2, 3, and Tables 5, 6 in Supplementary File 2). To gain a better understanding of the changes in functional connectivity between these two brain regions and the whole brain, functional connectivity was explored. The results indicated no statistically significant differences in the main effects, inter-group effects, and intra-group effects of CON and DMS for left orbital inferior frontal and left insula after SD and sleep recovery (details see Supplementary File 4).

### Mediator effect analysis

To better elucidate the relationship between magnetic stimulation and heart sound and nuclear magnetic indicators, and to verify whether magnetic stimulation has an impact on heart sound and brain region, the mediation effect analysis was performed to explore whether magnetic stimulation regulates the heart through affecting brain region, and stepwise regression for analysis was used (Wen Zhonqlin, 2014)

### Correlation analysis

**FIGURE 6:**
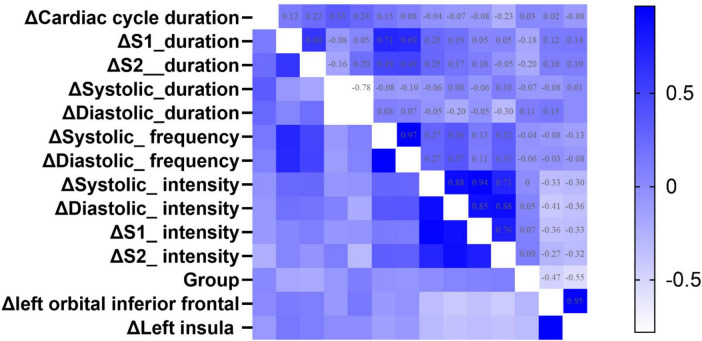
The heatmap of Spearman correlation analysis between group and the changed value of fMRI, heart sound indexes. **Δ** Means Gain value between SD 36 h and RS. The color represents the strength of the correlation coefficient. A deeper blue color indicates a greater positive correlation coefficient, while a deeper white color indicates a greater negative correlation coefficient.

Figure 6 illustrates the results of Spearman correlation analysis of gain values. Δ Left orbital inferior frontal was negatively correlated with Δ Systolic_intensity (rho = −0.33, *p* < 0.05), Δ Diastolic_intensity (rho = −0.41, *p* < 0.05), Δ S1_intensity (rho = −0.36, *p* < 0.05), and Δ S2_intensity (rho = −0.43, *p* < 0.05). Δ Left insula was negatively correlated with Δ Diastolic_intensity (rho = −0.36, *p* < 0.05), Δ S1_intensity (rho = −0.33, *p* < 0.05), and Δ S2_intensity (rho = −0.36, *p* < 0.05). Group was negatively correlated with Δ S2_duration (rho = −0.20, *p* < 0.05), Δ left orbital inferior frontal (rho = −0.27, *p* < 0.05), and Δ left insula (rho = −0.32, *p* < 0.05) (details see Table 4 in Supplementary File 2).

**TABLE 4:** The result of stepwise regression between Δ left orbital inferior frontal, group, and heart index.

Variable	Δ Left orbital inferior frontal	Δ s2_duration			Δ s1_intensity			Δ s2_intensity			Δ Systolic_intensity			Δ Diastolic_intensity		
		**Step 1 (β)**	**Step 2 (β)**	**Step 3 (β)**	**Step 1 (β)**	**Step 2 (β)**	**Step 3 (β)**	**Step 1 (β)**	**Step 2 (β)**	**Step 3 (β)**	**Step 1 (β)**	**Step 2 (β)**	**Step 3 (β)**	**Step 1 (β)**	**Step 2 (β)**	**Step 3 (β)**
Group	−0.519**	−0.049*		−0.052*	0.128		−0.219	0.199		−0.234	0.047		−0.207	0.078		−0.215
Δ Left orbital inferior frontal			0.018	−0.005		−0.571	−0.669		−0.730**	−0.835**		−0.396	−0.488		−0.468*	−0.564*
*F*	10.58**	5.85*	0.800	2.880	0.120	2.980	1.600	0.400	7.570	3.99*	0.030	2.850	1.660	0.100	4.96*	2.800
*R* ^2^	0.210	0.143	0.022	0.145	0.003	0.079	0.086	0.011	0.178	0.190	0.001	0.075	0.089	0.003	0.124	0.142

*p < 0.05;

**p < 0.01.

### Two-step regression analysis and mediator effect analysis

**FIGURE 7:**
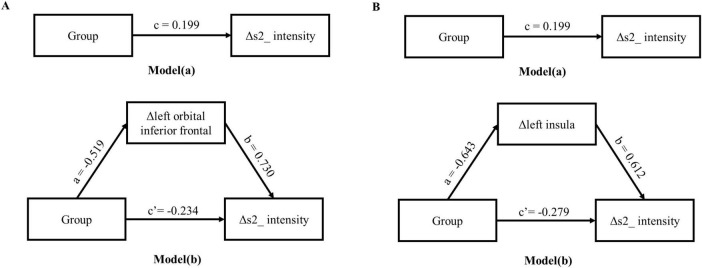
Intermediate effects test model path diagram. **(A)** The mediating model of group, Δ left orbital inferior frontal and Δ s2_intensity; **(B)** the mediating model of group, Δ left insula and Δ s2_intensity.

Combined with the correlation analysis, there was a pairwise correlation between group, heart sound index, and fMRI index. Therefore, the heart sound index defined as the dependent variable, and group and fMRI index as the independent variable to establish a stepwise regression model. The results showed that the regression models of Δ s2_intensity, group, and Δ left orbital inferior frontal (*F* = 3.99, *p* < 0.05) and Δ left insula (*F* = 3.3, *p* < 0.05) were meaningful (Tables 4, 5). The intermediate effect was tested by Bootstrap sampling method. According to the stepwise regression improvement method of WEN (Wen Zhonqlin, 2014), the steps were analyzed as follows: in the Δ s2_intensity, group, and Δ left orbital inferior frontal model (Figure 7A and Table 4), the total effect c was 0.199 (*p* > 0.05). The indirect effects were *a* = −0.519 (*p* < 0.01) and *b* = 0.730 (*p* < 0.01) respectively, so the indirect effects were significant. After testing with Bootstrap method, the confidence interval [0.047–0.820] did not include 0 (Table 6), and the direct effect *c* = −0.234 (*p* > 0.05), so there was only an intermediate effect. Also, there were only mediating effects in the Δ s2_intensity, group and Δ left insula model (Figure 7B and Tables 5, 7), respectively. It was suggested that the DMS can affect heart working load through the mediating effect via stimulation on specific brain areas.

**TABLE 5:** The result of stepwise regression between Δ left insula, group, and heart index.

Variable	Δ Left insula	Δ s2_duration			Δ s1_intensity			Δ s2_intensity			Δ Systolic_intensity			Δ Diastolic_intensity		
		**Step 1 (β)**	**Step 2 (β)**	**Step 3 (β)**	**Step 1 (β)**	**Step 2 (β)**	**Step 3 (β)**	**Step 1 (β)**	**Step 2 (β)**	**Step 3 (β)**	**Step 1 (β)**	**Step 2 (β)**	**Step 3 (β)**	**Step 1 (β)**	**Step 2 (β)**	**Step 3 (β)**
Group	−0.643***	−0.049*		−0.056*	0.128		−0.246	0.199		−0.279	0.047		−0.232	0.078		−0.237
Δ Left insula			0.016	−0.010		−0.465	−0.581		−0.612*	−0.744*		−0.324	−0.433		−0.377	−0.489*
*F*	15.24***	5.85*	0.750	2.980	0.120	2.280	1.280	0.400	6.03*	3.3*	0.030	2.200	1.370	0.100	3.670	2.190
*R* ^2^	0.303	0.143	0.021	0.149	0.003	0.061	0.070	0.011	0.147	0.163	0.001	0.059	0.075	0.003	0.095	0.114

*p < 0.05;

***p < 0.001.

**TABLE 6:** The bootstrap mediation effect of left orbital inferior frontal on group and heart sound.

Path	Effect	*p*	Bootstrap 95% CI	
DMS-Δ left orbital inferior frontal-Δ s2_duration	0.003	0.792	−0.017	0.022
DMS-Δ left orbital inferior frontal-Δ Systolic_intensity	0.253	0.109	−0.056	0.563
DMS-Δ left orbital inferior frontal-Δ Diastolic_intensity	0.293	0.061	−0.014	0.599
DMS-Δ left orbital inferior frontal-Δ s1_intensity	0.347	0.038	0.019	0.675
DMS-Δ left orbital inferior frontal-Δ s2_intensity	0.433	0.028	0.047	0.820

**TABLE 7:** The bootstrap mediation effect of left insula on group and heart sound.

Path	Effect	*p*	Bootstrap 95% CI	
DMS-Δ left insula-Δ s2_duration	0.007	0.600	−0.018	0.031
DMS-Δ left insula-Δ Systolic_intensity	0.278	0.098	−0.051	0.608
DMS-Δ left insula-Δ Diastolic_intensity	0.314	0.070	−0.026	0.655
DMS-Δ left insula-Δ s1_intensity	0.374	0.065	−0.023	0.770
DMS-Δ left insula-Δ s2_intensity	0.479	0.027	0.055	0.902

## Discussion

To the best of our knowledge, this study represents the first attempt to integrate heart sound and fMRI data to validate the impact of DMS intervention post SD. Notably, previous studies have demonstrated the potential of DMS intervention to promote recovery in patients with cardiovascular or lung diseases by reducing heart rate and increasing HRV, thereby reducing cardiac workload and enhancing blood oxygen uptake (Zhong et al., 2005), besides, in the study of the effect of bilateral stimulation of the subthalamic nucleus on heart rate variability in patients with Parkinson’s disease (PD), it was found that DMS may enhance sympathetic regulation and thus affect heart rate (Liu et al., 2013). Our findings revealed the significant differences in heart sound parameters between the DMS and CON groups after SD. Specifically, the DMS group exhibited a significantly longer heart cycle duration, decreased heart sound frequency, and lower heart sound intensity compared to the CON group. Physiologically, the intensity and frequency of heart sounds, along with their interrelationship, can serve as indicators of cardiac valve function, myocardial performance, and cardiac blood flow status (Fukuda et al., 1981). This evidence suggests that DMS intervention may mitigate the accumulated negative effects on the cardiac-brain axis to some extent, possibly by enhancing sympathetic regulation (Liu et al., 2013), or by activating specific brain regions.

Regarding the specific brain areas affected by DMS post-SD, we observed a significant reduction in the DC of the orbitofrontal gyrus and insula. The insula is part of the salience network, crucial for processing sensorimotor information, general cognition, and coordinating emotions, pain, and physical movement. The orbital frontal gyrus is implicated in cognitive function following SD. Our findings suggested that DMS-induced neural modulation may contribute to improved recognition of error information, as evidenced by changes in the connectivity properties of the insula in the resting brain functional network (Ham et al., 2013) Furthermore, the functional connection results indicated that the connectivity between these brain regions and the whole brain did not change significantly over time, suggesting that DMS failed to induce alterations in the functional connectivity of these regions in a short treatment period. This implies that a brief night of restorative sleep or a single 30-min magnetic stimulation may not fully restore the connectivity between these regions and other brain areas, possibly indicating the irreversibility of 36 h of SD in this context (Sun et al., 2022). In previous studies, DMS could slow the progression of early resting tremor in PD (Hacker et al., 2018) and was a well-established treatment for PD (Habets et al., 2018). Although theoretically, DMS should enhance functional connectivity after SD, which may be related to the stimulation parameters. Furthermore, short-term interventions often fail to elicit substantial changes in neuroplasticity (De Gennaro et al., 2007). It may be necessary to extend the duration of restorative sleep or increase the duration and quantity of magnetic stimulation to further investigate the effects of intervention measures on the recovery of brain functional connectivity.

Delving further into our investigation, we explored the correlations among DMS indices, heart sound characteristics, and fMRI data, the findings revealed the significant associations. Notably, the DC gain values of the left orbital inferior frontal gyrus and insula exhibited negative correlations with the intensity of systolic and diastolic heart sounds. Additionally, the group variable presented the negative correlations with S2 duration, left orbital inferior frontal gyrus, and left insula. These findings align with Lutz et al.’s (2009) work, demonstrating a distinct correlation between the BOLD signal in the insula and heart function, specifically HR, underscoring the intricate relationship between the insula and heart function. Furthermore, a study on the temporal and spatial characteristics of brain response and heart activity during fear image processing disclosed increased HR responses to stimuli related to arachnophobia, associating with heightened insula and hippocampus activity (Michalowski et al., 2017).

**FIGURE 8:**
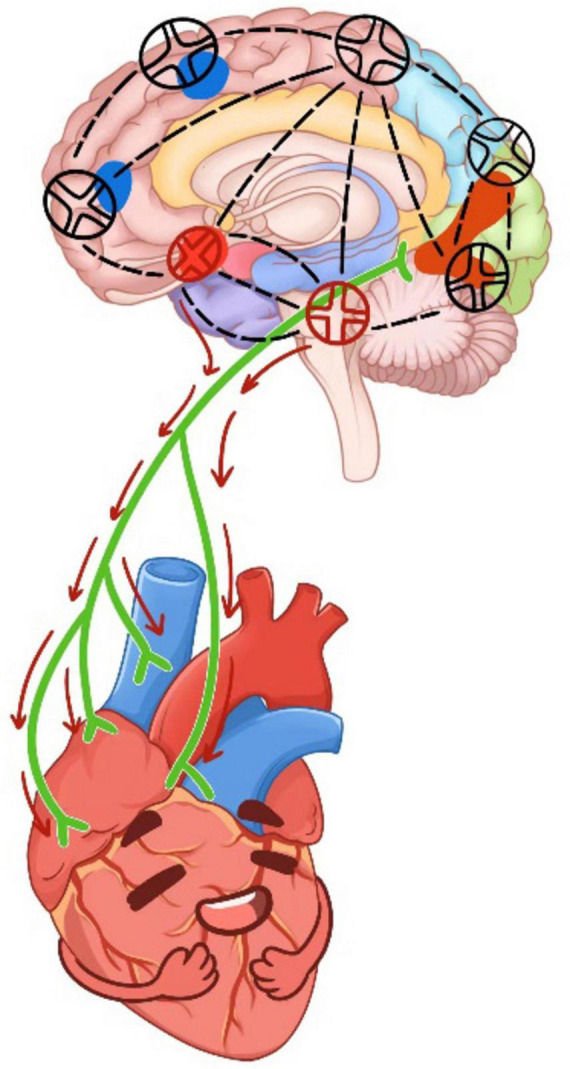
The working principle of DMS on cardiac-brain axis. The red circle was located in the left orbitofrontal gyrus and insula.

Combined with the mediating effect results, it was shown that DMS affected s2_intensity by intervening in brain regions. Previous studies suggested that the cardiac cycle was a core element of the heart-brain axis that regulates perception, cognition, emotional processing, and action (Schulz et al., 2009; Gray et al., 2012; Garfinkel et al., 2014; Barrett and Simmons, 2015; Critchley and Garfinkel, 2017; Candia-Rivera et al., 2024; Lai et al., 2024). This regulation is thought to be mediated in part by the multi-synaptic ascending pathway produced by aortic baroreceptors (Edwards et al., 2002). Studies shown that heartbeat-induced pulsations of cerebral blood vessels can directly affect central neuronal activity through the activation of mechanosensitive channels (Jammal Salameh et al., 2024). In this study, it was found that DMS affected the orbitofrontal gyrus and insula to regulate the intensity of S2 heart sounds (see Figure 8).

Therefore, it was possible that the DMS regulated the neuronal conduction of orbitofrontal gyrus and insula, causing changes in central neurons and affecting arterial pressure receptors, thereby reducing the intensity of s2 heart sounds (Rodriguez-Romaguera et al., 2015). The findings further confirmed the mediating role of brain regions from the side. Several similar results have been found in previous studies, which validated that certain brain regions, including the left supraorbital frontal gyrus, left infraorbital frontal gyrus, and right posterior cingulate gyrus, in patients with chronic heart failure and cognitive impairment exhibited less centrality compared to those without cognitive impairment. Impaired brain network properties and reduced connectivity were identified as features of progressive disruption of brain networks, predicting the development of cognitive impairment in patients with chronic heart failure (Wang et al., 2023). Additionally, a positive correlation between cortical thickness of the suborbital gyrus and respiratory sinus arrhythmia was observed (Korom et al., 2023). In a meta-analysis, non-invasive brain stimulation was found to effectively influence cardiovascular and autonomic nervous system activity (Makovac et al., 2017). Our study validates the detrimental effects of SD, which may significantly disrupt the cardiac-brain axis (Manea et al., 2015). In contrast, the non-invasive intervention of DMS demonstrates efficacy in mitigating the impact post-SD.

Combined with the current results, even though the response to intervention elucidated partly, the specific neuromodulation mechanism remains unclear. The appropriate animal model can be performed to compare the neurotransmitters dynamic regulation in cardiac brain axis between pre and post intervention. While the appropriate stimulation parameters and specific personal medical background need to balance the benefit and risk.

## Conclusion

Sleep deprivation significantly impacts cardiac-brain axis function. Non-invasive DMS intervention appears effective in reducing activity in the orbitofrontal gyrus and insula and increasing cardiac cycle duration, potentially aiding recovery from SD.

### Limitation

The indices objectively reflected the changes during SD but lack specific outlier ranges. Normal heart sounds during sleep were not collected due to ethic restriction. This study included only male individuals, it may introduce the potential sex bias. Since this was an exploratory pilot study, the endpoint index was relatively broad and the sample size was not calculated.

## Data Availability

The raw data supporting the conclusions of this article will be made available by the authors, without undue reservation.
