# Supplementary material for: The effect of deep magnetic stimulation on the cardiac-brain axis post-sleep deprivation: a pilot study
Source: Front Neurosci. 2025 Jan 10;18:1464299. doi: 10.3389/fnins.2024.1464299 (PMC11757894; doi:10.3389/fnins.2024.1464299)
Supplement: Supplementary file 1 [file Data_Sheet_1.docx]

**Heart sound data acquisition and analysis process**

**1. Collection of heart sound data**

**1.1Acquisition location**

Mitral valve area (the first auscultation area), aortic valve area (the second auscultation area), main A valve (the third auscultation area), pulmonary valve area (the fourth auscultation area) and tricuspid valve area (the fifth auscultation area)

**1.2 Acquisition process**

The data were collected via the ETZ-1A(C) electronic stethoscope。The steps are as follows :(1) After disinfecting the stethoscope before use, stick the probe on the palm for 1-2 minutes. (2) After surrounding silence, subjects exposed their chest and took supine, lateral or sitting position. (3) Then insert the auscultation head into the headphone jack of the mobile phone (some models require a converter). (4) Then press the auscultation head on the five valve areas successively, after 5 seconds and turn on the mobile phone recorder for recording. (5) Finally, the test subjects saved the audio named after the heart sound record as the date-student number-auscultation area. Each subject was collected every 2 hours, 60s for each auscultation area.

**2. Data extraction process**

**2.1 Software**

Used Audacity software (version: 1.3.3)^1^ to evaluate the heart sounds collected by the stethoscope.

**2.2 Characteristic**

The indicators analyzed by the software are shown as follow.

**2.2.1 Duration(unit: seconds):**

**S1_duration,**Reflecting the tricuspid valve status and the blood volume of the left ventricle, a sign of incipient cardiac failure^2^;

**S2_duration,** it reflects the function of semilurar valve and the pressure of aorta and pulmonary artery;

**Systolic_duration,** reflecting the contractile function of the ventricular muscle;

**Diastolic_duration,** reflecting the ventricular diastolic function.

**2.2.2Intensity(unit: dB):**

**S1_Intensity**(s1_maxdb;s1_mindb;s1_meandb;s1_middledb) , reflecting the tricuspid valve status and the blood volume of the left ventricle ，negative correlation between the S(1) intensity and the area of calcification^3^;

**S2_ Intensity**(s2_maxdb;s2_mindb;s2_meandb; s2_middledb):reflecting the aortic and pulmonary valve conditions and arterial pressure;

**Systoli_Intensity**(systolic_maxdb;systolic_mindb;systolic_meandb;systolic_middledb;systolic_absomean;systolic_std;systolic_skew;systolic_kurt;systolic_max;systolic_min;systolic_peak2valley;systolic_rms;systolic_crestfactor;systolic_shapefactor;systolic_impulsefactor;systolic_marginfactor;systolic_energy): Grading and recognition of a systolic murmur**;**

**Diastolic**_**Intensity**(Diastolic_maxdb;Diastolic_mindb;Diastolic_meandb;Diastolic_middledb;Diastolic_absomean;Diastolic_std;Diastolic_skew;Diastolic_kurt;Diastolic_max

Diastolic_min;Diastolic_peak2valley;Diastolic_rms;Diastolic_crestfactor;Diastolic_shapefactor;Diastolic_impulsefactor;Diastolic_marginfactor;Diastolic_energy),Identifying the presence of heart failure: the third and fourth heart sounds, recognition of a diastolic period murmur.

**2.2.3Frequency(unit: Hz)**

**Systoli_Frequency**(systolic_first_f0;systolic_middle_f0;systolic_last_f0;systolic_median_f0;systolic_mean_f0;systolic_f0variation;systolic_f0skew;systolic_f0kurt;systolic_max_f0;systolic_min_f0;systolic_range_f0;systolic_slope_start2max;systolic_slope_max2end;systolic_hnr;systolic_jitter;systolic_hr_mean;systolic_hr_median;systolic_hr_std;systolic_hr_max**;**systolic_hr_min),ventricular contractility: identifying heart failure, peripheral resistance status**.**

**Diastolic**_**Frequency**(Diastolic_first_f0;Diastolic_middle_f0;Diastolic_last_f0;Diastolic_median_f0;Diastolic_mean_f0;Diastolic_f0variation;Diastolic_f0skew;Diastolic_f0kurt;Diastolic_max_f0;Diastolic_min_f0;Diastolic_range_f0;Diastolic_slope_start2max;Diastolic_slope_max2end;Diastolic_hnr;Diastolic_jitter;Diastolic_hr_mean;Diastolic_hr_median;Diastolic_hr_std;Diastolic_hr_max;Diastolic_hr_min), cardiac tumor condition, cardiac souffle**.**

**3. The process of dimensionality reduction analysis of heart sound data**

**3.1Duration**

Each stage has only one index, which is directly used as the analysis index

**3.2Intensity and Frequency indicators dimension reduction process**

**3.2.1 KMO test**

KMO(Kaiser-Meyer-Olkin) test statistic^4^ is used to compare simple correlation coefficient and partial correlation coefficient between variables. The purpose of KMO test and Bartlett sphericity test is to determine whether the data is suitable for principal component analysis. The KMO statistic is between 0 and 1. Kaiser provides a common KMO metric :Be very suitable for factor analysis Greater than 0.9, 0.8 ~ 0.9 means very suitable for factor analysis, 0.7 ~ 0.8 means suitable for factor analysis, 0.6 ~ 0.7 means barely fit factor analysis. And the Bartlett sphericity test P-value should be less than 0.05.

The results of KMO test and Bartlett test of sphericity show that the intensity and frequency of each heart sound segment can be used for principal component analysis(Table 1).

Table 2 KMO and Bartlett test results

| Vari2able | Test method | Index | Test result |
| --- | --- | --- | --- |
| Intensity_S1 | KMO test | KMO | 0.637 |
|  | Bartlett test of sphericity | Chi-Square | 2.85e+05 |
|  |  | df | 6 |
|  |  | Sig. | 0.000 |
| Intensity_S2 | KMO test | KMO | 0.635 |
|  | Bartlett test of sphericity | Chi-Square | 2.83e+05 |
|  |  | df | 6 |
|  |  | Sig. | 0.000 |
| Intensity_Systolic | KMO test | KMO | 0.849 |
|  | Bartlett test of sphericity | Chi-Square | 7.87e+05 |
|  |  | df | 136 |
|  |  | Sig. | 0.000 |
| Intensity_Diastolic | KMO test | KMO | 0.860 |
|  | Bartlett test of sphericity | Chi-Square | 8.04e+05 |
|  |  | df | 136 |
|  |  | Sig. | 0.000 |
| Frequency_Systolic | KMO test | KMO | 0.863 |
|  | Bartlett test of sphericity | Chi-Square | 4.84e+05 |
|  |  | df | 190 |
|  |  | Sig. | 0.000 |
| Frequency_Diastolic | KMO test | KMO | 0.854 |
|  | Bartlett test of sphericity | Chi-Square | 5.14e+05 |
|  |  | df | 190 |
|  |  | Sig. | 0.000 |

**3.2.2 Principal component analysis**

Principal components were extracted according to eigenvalue and cumulative variance contribution rate^5^. Principal components with eigenvalue greater than 1 were extracted, and the cumulative variance contribution rate of extracted principal components was tried to be large. The number of extracted principal components was judged by the scree map(Figure 1).


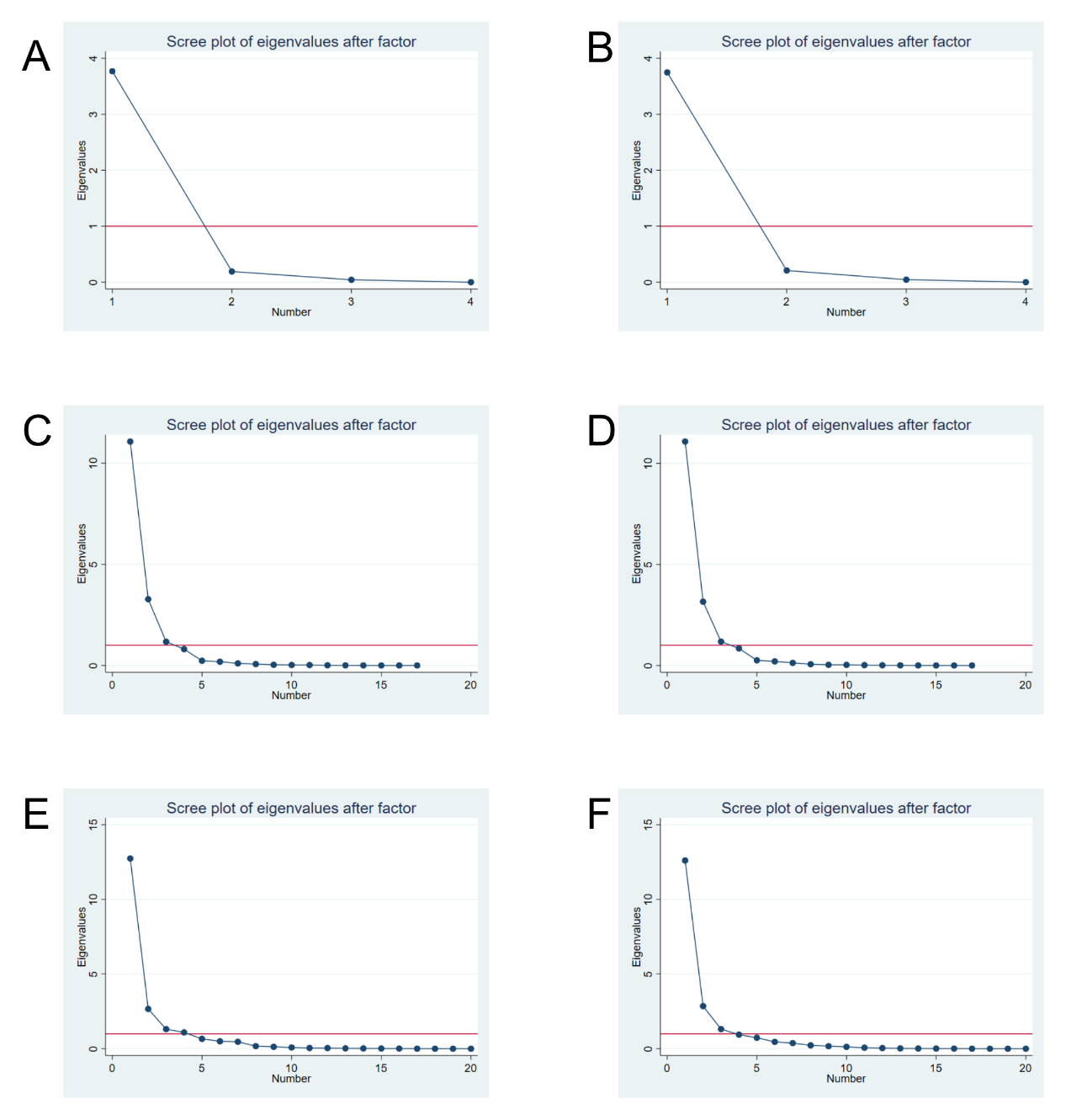


Figure 1 Each feature principal component analysis scree map. **A**. Intensity_S1 **B**. Intensity_S2 **C**. Intensity_Systolic **D**. Intensity_Diastolic **E**. Frequency_Systolic **F**. Frequency_Diastolic

**3.2.3 Generate composite index**

The comprehensive index is calculated according to the variance contribution rate of the extracted principal component, and the specific results are as follows(Table 3-8):

①Intensity_S1

The Intensity_S1 comprehensive index is calculated as: Intensity_S1=Factor1/0.9418(Table 3).

| Table 3 Intensity_S1 principal component analysis results | | | | |
| --- | --- | --- | --- | --- |
| Factor | Eigenvalue | Difference | Proportion | Cumulative |
| Factor1 | 3.76737 | 3.57681 | 0.9418 | 0.9418 |

②Intensity_S2

The Intensity_S2 comprehensive index is calculated as: Intensity_S2=Factor1/0.9368 (Table 4).

| Table 4 Intensity_S2 principal component analysis results | | | | |
| --- | --- | --- | --- | --- |
| Factor | Eigenvalue | Difference | Proportion | Cumulative |
| Factor1 | 3.74707 | 3.53888 | 0.9368 | 0.9368 |

③Intensity_Systolic

The Intensity_Systolic comprehensive index is calculated as: Intensity_S1= Intensity_Systolic=(Factor1*0.6511+Factor2*0.1926+Factor3*0.0688)/0.9126

(Table 5).

| Table 5 Intensity_Systolic principal component analysis results | | | | |
| --- | --- | --- | --- | --- |
| Factor | Eigenvalue | Difference | Proportion | Cumulative |
| Factor1 | 11.06823 | 7.79321 | 0.6511 | 0.6511 |
| Factor2 | 3.27502 | 2.10485 | 0.1926 | 0.8437 |
| Factor3 | 1.17017 | 0.36399 | 0.0688 | 0.9126 |

④Intensity_Diastolic

The Intensity_Diastolic comprehensive index is calculated as: Intensity_Diastolic=(Factor1*0.6519+Factor2*0.1856+Factor3*0.0694)/0.9069

(Table 6).

| Table 6 Intensity_Diastolic principal component analysis results | | | | |
| --- | --- | --- | --- | --- |
| Factor | Eigenvalue | Difference | Proportion | Cumulative |
| Factor1 | 11.08167 | 7.92689 | 0.6519 | 0.6519 |
| Factor2 | 3.15478 | 1.97414 | 0.1856 | 0.8374 |
| Factor3 | 1.18063 | 0.33767 | 0.0694 | 0.9069 |

⑤Frequency_Systolic

The Frequency_Systolic comprehensive index is calculated as: Frequency_Systolic=(Factor1*0.6372+Factor2*0.1332+Factor3*0.0656+Factor4*0.0546)/0.8906(Table 7).

| Table 7 Frequency_Systolic principal component analysis results | | | | |
| --- | --- | --- | --- | --- |
| Factor | Eigenvalue | Difference | Proportion | Cumulative |
| Factor1 | 12.74438 | 10.08024 | 0.6372 | 0.6372 |
| Factor2 | 2.66413 | 1.3514 | 0.1332 | 0.7704 |
| Factor3 | 1.31274 | 0.22115 | 0.0656 | 0.8361 |
| Factor4 | 1.09158 | 0.43113 | 0.0546 | 0.8906 |

⑥Frequency_Diastolic

The Frequency_Diastolic comprehensive index is calculated as: Frequency_Diastolic=(Factor1*0.6305+Factor2*0.1427+Factor3*0.0658)/0.8390(Table 8).

| Table 8 Frequency_Diastolic principal component analysis results | | | | |
| --- | --- | --- | --- | --- |
| Factor | Eigenvalue | Difference | Proportion | Cumulative |
| Factor1 | 12.61087 | 9.75634 | 0.6305 | 0.6305 |
| Factor2 | 2.85453 | 1.53896 | 0.1427 | 0.7733 |
| Factor3 | 1.31557 | 0.37181 | 0.0658 | 0.8390 |

Reference

1. Vidhya BA-O, Nikhil Madhav M, Suresh Kumar M, Kalanandini S. AI Based Diagnosis of Pneumonia. *Wirel Pers, Commun*, (2022).

2. Riecanský I Fau - Haviar V, Haviar V Fau - Plachá L, Plachá L. The first heart sound abnormalities accompanied with alteration of systolic time intervals in incipient heart failure. *Cor, Vasa*, (1979).

3. Mularek-Kubzdela T Fau - Grajek S*, et al.* First heart sound and opening snap in patients with mitral valve disease. Phonocardiographic and pathomorphologic study. *Int, J Cardiol*, (2008 Apr 25).

4. Zoch-Lesniak B*, et al.* Performance Measures for Short-Term Cardiac Rehabilitation in Patients of Working Age: Results of the Prospective Observational Multicenter Registry OutCaRe. *Arch Rehabil Res Clin, Transl*, (2020 Jun).

5. Ben Salem K Fau - Ben Abdelaziz A, Ben Abdelaziz A. Principal Component Analysis (PCA). *Tunis, Med*, (2021 Avril).
