# Supplementary material for: The effect of deep magnetic stimulation on the cardiac-brain axis post-sleep deprivation: a pilot study
Source: Front Neurosci. 2025 Jan 10;18:1464299. doi: 10.3389/fnins.2024.1464299 (PMC11757894; doi:10.3389/fnins.2024.1464299)
Supplement: Supplementary file 2 [file Data_Sheet_2.docx]

**Supplementary file 2**

**Supplementary form for heart Sound Analysis:**

1. **Comparison between two groups**

At SD36H, The difference of s1_duration, Systolic_ intensity, Diastolic_ intensity, s1_ intensity and s2_ intensity the between two groups were statistically significant(see *p^a^).* The paired T-test results showed that only s2__duration was statistically significant in the DMS group before and after intervention, and s2__duration was extended after RS (see *p^b^*). The results of paired T-test showed that there was no significant difference in the heart sound data of CON group before and after natural sleep (see *p^c^*). The change data shows that only Δs2__duration was statistically significant between two groups, and Δs2__duration was extended compared with CON group(see *p^b^*).

| **Supplementary file 2 Table 1 Changes in the heart sound between before and after intervention. *(Mean ± SD)*** | | | | | | | | |
| --- | --- | --- | --- | --- | --- | --- | --- | --- |
| Heart sound | SD36H | | *p^a^* | RS | | *p^b^* | *p^c^* | *p^d^* |
|  | DMS group | CON group |  | DMS group | CON group |  |  |  |
| Cardiac cycle duration | 0.81±0.04 | 0.79±0.04 | 0.05 | 0.81±0.04 | 0.80±0.03 | 0.53 | 0.6 | 0.96 |
| S1_duration | 0.13±0.03 | 0.16±0.05 | 0.01 | 0.15±0.05 | 0.15±0.05 | 0.17 | 0.31 | 0.09 |
| S2__duration | 0.12±0.04 | 0.14±0.04 | 0.11 | 0.15±0.05 | 0.15±0.04 | 0.03 | 0.19 | 0.02 |
| Systolic_duration | 0.40±0.06 | 0.39±0.05 | 0.78 | 0.43±0.07 | 0.40±0.04 | 0.13 | 0.72 | 0.43 |
| Diastolic_duration | 0.42±0.06 | 0.40±0.05 | 0.23 | 0.38±0.06 | 0.40±0.04 | 0.05 | 0.45 | 0.38 |
| Systolic_ frequency | -0.07±0.56 | 0.24±0.56 | 0.06 | -0.16±0.77 | 0.31±0.45 | 0.84 | 0.9 | 0.95 |
| Diastolic_ frequency | -0.07±0.57 | 0.25±0.56 | 0.05 | -0.16±0.81 | 0.35±0.49 | 0.81 | 0.98 | 0.87 |
| Systolic_ intensity | -0.34±0.60 | 0.20±0.62 | 0.00 | -0.28±0.60 | 0.61±0.75 | 0.41 | 0.45 | 0.86 |
| Diastolic_ intensity | -0.35±0.62 | 0.19±0.55 | 0.00 | -0.29±0.58 | 0.62±0.75 | 0.28 | 0.32 | 0.75 |
| S1_ intensity | -0.44±0.87 | 0.29±0.82 | 0.00 | -0.43±1.04 | 0.77±0.76 | 0.77 | 0.42 | 0.73 |
| S2_ intensity | -0.41±0.84 | 0.20±0.75 | 0.01 | -0.37±0.87 | 0.70±0.75 | 0.46 | 0.19 | 0.53 |

*Note: DMS, deep magnetic stimulation; CON, control; RS, recovery sleep; SD36H: sleep deprivation 36 hours*

*p^a^:SD36H between the groups. p^b^: The difference in the DMS group before and after the intervention. p^c^: The variation in the control group before and after the intervention. p^d^: change vales between the groups. Δ means change data.*

1. **Two-factor analysis**

The two-factor analysis results revealed a significant group effect on cardiac cycle duration, systolic frequency, diastolic frequency, systolic intensity, diastolic intensity, S1 intensity, and S2 intensity. No interaction effect was observed. Main effect analysis indicated that the DMS group presented a significant longer cardiac cycle duration, lower heart tone frequency, and reduced heart sound intensity compared to the CON group (Supplementary file 2 Table 2)

| **Supplementary file 2 Table 2 Results of repeated two-factor measurements of heart sound by ANOVA/nonparametric two-factor analysis** | | | |
| --- | --- | --- | --- |
| Heart sound | | F/H | P |
| Cardiac cycle duration | group | 6.01 | 0.02 |
|  | status | 0.32 | 0.57 |
|  | Session#Group | 0.28 | 0.60 |
| s1_duration | group | 2.97 | 0.09 |
|  | status | 0.94 | 0.34 |
|  | Session#Group | 1.90 | 0.17 |
| s2__duration | group | 0.99 | 0.32 |
|  | status | 3.83 | 0.05 |
|  | Session#Group | 0.90 | 0.35 |
| systolic_duration | group | 2.35 | 0.13 |
|  | status | 3.37 | 0.07 |
|  | Session#Group | 1.35 | 0.25 |
| diastolic_duration | group | 0.03 | 0.86 |
|  | status | 2.26 | 0.14 |
|  | Session#Group | 2.55 | 0.11 |
| Systolic_ frequency | group | 9.66 | <0.01 |
|  | status | 0.00 | 0.96 |
|  | Session#Group | 0.39 | 0.53 |
| Diastolic_ frequency | group | 10.62 | <0.01 |
|  | status | 0.00 | 0.97 |
|  | Session#Group | 0.59 | 0.44 |
| Systolic_ intensity | group | 29.06 | <0.01 |
|  | status | 2.98 | 0.09 |
|  | Session#Group | 1.67 | 0.20 |
| diastolic_ intensity | group | 30.81 | <0.01 |
|  | status | 3.53 | 0.06 |
|  | Session#Group | 1.99 | 0.16 |
| s1_ intensity | group | 27.73 | <0.01 |
|  | status | 1.78 | 0.19 |
|  | Session#Group | 1.63 | 0.20 |
| s2_ intensity | group | 25.31 | <0.01 |
|  | status | 2.61 | 0.11 |
|  | Session#Group | 1.91 | 0.17 |

Group: control group and DMS group

Session: include before and after recover sleep.

1. **Regression analysis**

The regression analysis results revealed a significant group effect on cardiac cycle duration, s1__duration, systolic intensity, diastolic intensity, S1 intensity, and S2 intensity. No interaction effect was observed. Group effect analysis indicated that the DMS group presented a significant longer cardiac cycle duration, and reduced s1__duration and heart sound intensity compared to the CON group (Supplementary file 2 Table 3)

| **Supplementary file 2 Table 3 Regression analysis of Heart sounds indicators by group and session** | | | | | | |
| --- | --- | --- | --- | --- | --- | --- |
|  |  |  | *β* | Std. Err | t | p |
| cardiac cycle duration | Group(Compare with DMS group) | CON group | -0.02 | 0.01 | -2.15 | 0.03 |
|  | Session (Compare with SD36H) | RS | 0.00 | 0.01 | 0.03 | 0.98 |
|  | Session#Group |  | 0.01 | 0.02 | 0.53 | 0.60 |
| s1__duration | Group(Compare with DMS group) | CON group | 0.03 | 0.01 | 2.24 | 0.03 |
|  | Session (Compare with SD36H) | RS | 0.02 | 0.01 | 1.73 | 0.09 |
|  | Session#Group |  | -0.03 | 0.02 | -1.38 | 0.17 |
| s2__duration | Group(Compare with DMS group) | CON group | 0.02 | 0.01 | 1.40 | 0.17 |
|  | Session (Compare with SD36H) | RS | 0.03 | 0.01 | 2.15 | 0.04 |
|  | Session#Group |  | -0.02 | 0.02 | -0.95 | 0.35 |
| systolic_duration | Group(Compare with DMS group) | CON group | 0.00 | 0.02 | -0.27 | 0.79 |
|  | Session (Compare with SD36H) | RS | 0.04 | 0.02 | 2.21 | 0.03 |
|  | Session#Group |  | -0.03 | 0.02 | -1.16 | 0.25 |
| diastolic_duration | Group(Compare with DMS group) | CON group | -0.02 | 0.02 | -1.28 | 0.20 |
|  | Session (Compare with SD36H) | RS | -0.04 | 0.02 | -2.29 | 0.02 |
|  | Session#Group |  | 0.04 | 0.02 | 1.60 | 0.11 |
| Systolic_ frequency | Group(Compare with DMS group) | CON group | 0.31 | 0.17 | 1.79 | 0.08 |
|  | Session (Compare with SD36H) | RS | -0.08 | 0.17 | -0.50 | 0.62 |
|  | Session#Group |  | 0.16 | 0.25 | 0.63 | 0.53 |
| Diastolic_ frequency | Group(Compare with DMS group) | CON group | 0.32 | 0.18 | 1.79 | 0.08 |
|  | Session (Compare with SD36H) | RS | -0.09 | 0.17 | -0.54 | 0.59 |
|  | Session#Group |  | 0.20 | 0.26 | 0.77 | 0.44 |
| Systolic_ intensity | Group(Compare with DMS group) | CON group | 0.55 | 0.18 | 2.96 | <0.01 |
|  | Session (Compare with SD36H) | RS | 0.06 | 0.18 | 0.32 | 0.75 |
|  | Session#Group |  | 0.34 | 0.27 | 1.29 | 0.20 |
| diastolic_ intensity | Group(Compare with DMS group) | CON group | 0.54 | 0.18 | 2.99 | <0.01 |
|  | Session (Compare with SD36H) | RS | 0.06 | 0.18 | 0.34 | 0.73 |
|  | Session#Group |  | 0.37 | 0.26 | 1.41 | 0.16 |
| s1_ intensity | Group(Compare with DMS group) | CON group | 0.73 | 0.25 | 2.88 | 0.01 |
|  | Session (Compare with SD36H) | RS | 0.01 | 0.25 | 0.04 | 0.97 |
|  | Session#Group |  | 0.47 | 0.37 | 1.28 | 0.21 |
| s2_ intensity | Group(Compare with DMS group) | CON group | 0.61 | 0.23 | 2.63 | 0.01 |
|  | Session (Compare with SD36H) | RS | 0.04 | 0.23 | 0.17 | 0.86 |
|  | Session#Group |  | 0.46 | 0.33 | 1.38 | 0.17 |

1. **Correlation analysis**

Δ Left orbital inferior frontal was negatively correlated with Δ Systolic_intensity (rho = -0.33, p < 0.05), Δ Diastolic_intensity (rho = -0.41, p < 0.05), Δ S1_intensity (rho = -0.36, p < 0.05), and Δ S2_intensity (rho = -0.42, p < 0.05). Δ Left insula was negatively correlated with Δ Diastolic_intensity (rho = -0.36, p < 0.05), ΔS1_intensity (rho = -0.33, p < 0.05), and Δ S2_intensity (rho = -0.36, p < 0.05). Group was negatively correlated with ΔS2_duration (rho = -0.20, p < 0.05), Δ left orbital inferior frontal (rho = -0.27, p < 0.05), and Δ left insula (rho = -0.32, p < 0.05).

| **Supplementary file 2** Table 4 The results of the Pearson correlation analysis | | | | | | | | | | | | | | | |
| --- | --- | --- | --- | --- | --- | --- | --- | --- | --- | --- | --- | --- | --- | --- | --- |
|  |  | 1 | 2 | 3 | 4 | 5 | 6 | 7 | 8 | 9 | 10 | 11 | 12 | 13 | 14 |
| 1 | Δ cardiac cycle duration | 1 |  |  |  |  |  |  |  |  |  |  |  |  |  |
| 2 | Δ S1_duration | 0.12 | 1 |  |  |  |  |  |  |  |  |  |  |  |  |
| 3 | Δ S2__duration | 0.22 | 0.60* | 1 |  |  |  |  |  |  |  |  |  |  |  |
| 4 | Δ Systolic_duration | 0.33* | -0.08 | -0.16 | 1 |  |  |  |  |  |  |  |  |  |  |
| 5 | Δ Diastolic_duration | 0.24 | 0.05 | 0.20 | -0.78* | 1 |  |  |  |  |  |  |  |  |  |
| 6 | Δ Systolic_ frequency | 0.15 | 0.71* | 0.50* | -0.08 | 0.08 | 1 |  |  |  |  |  |  |  |  |
| 7 | Δ Diastolic_ frequency | 0.08 | 0.69* | 0.50* | -0.10 | 0.07 | 0.97* | 1 |  |  |  |  |  |  |  |
| 8 | ΔSystolic_ intensity | -0.04 | 0.25 | 0.25 | -0.06 | -0.05 | 0.27 | 0.27 | 1 |  |  |  |  |  |  |
| 9 | Δ Diastolic_ intensity | -0.07 | 0.19 | 0.17 | 0.08 | -0.20 | 0.36* | 0.37* | 0.88* | 1 |  |  |  |  |  |
| 10 | Δ S1_ intensity | -0.08 | 0.05 | 0.10 | -0.07 | -0.05 | 0.13 | 0.11 | 0.94* | 0.85* | 1 |  |  |  |  |
| 11 | Δ S2_ intensity | -0.23 | 0.05 | -0.05 | 0.10 | -0.30 | 0.32 | 0.31 | 0.71* | 0.88* | 0.76* | 1 |  |  |  |
| 12 | Δ left orbital inferior frontal | 0.02 | 0.12 | 0.10 | -0.08 | 0.15 | -0.08 | -0.03 | -0.33* | -0.41* | -0.36* | -0.42* | 1 |  |  |
| 13 | Δ left insula | -0.08 | 0.14 | 0.10 | 0.01 | 0.00 | -0.13 | -0.08 | -0.30 | -0.36* | -0.33* | -0.36* | 0.95* | 1 |  |
| 14 | group | 0.03 | -0.18 | -0.20* | -0.07 | 0.11 | -0.04 | -0.06 | 0.00 | 0.05 | 0.07 | 0.09 | -0.27* | -0.32* | 1 |

* The significance level of the mean difference is 0.05. Δ means change data.

**fMRI data supplement form:**

1. **DC Weight0.25**

**Supplementary file 2 Table 5 DC Weight0.25_The difference of interaction effect was statistically significant**

| Peak Location | Side | Cluster  Size | MNI  Coordinates | | | Peak  T score | P-value  corrected  by FWE |
| --- | --- | --- | --- | --- | --- | --- | --- |
|  |  |  | X | Y | Z |  |  |
| Insula_L | Left Cerebrum | 47 | -27 | 21 | -9 | 25.7476 | <0.0001 |

1. **DC Binary0.25**

**Supplementary file 2 Table 6 DC Binary0.25 _The difference of interaction effect was statistically significant**

| Peak Location | Side | Cluster  Size | MNI  Coordinates | | | Peak  T score | P-value  corrected  by FWE |
| --- | --- | --- | --- | --- | --- | --- | --- |
|  |  |  | X | Y | Z |  |  |
| Frontal_Inf_Orb_L | Left Cerebrum | 27 | -27 | 24 | -9 | 23.0116 | <0.0001 |
