# Supplementary material for: The effect of deep magnetic stimulation on the cardiac-brain axis post-sleep deprivation: a pilot study
Source: Front Neurosci. 2025 Jan 10;18:1464299. doi: 10.3389/fnins.2024.1464299 (PMC11757894; doi:10.3389/fnins.2024.1464299)
Supplement: Supplementary file 3 [file Data_Sheet_3.docx]

*supplementary file 3*

**Results of other auscultation areas**

**1.The second auscultation area**

The results of the two-factor analysis disclosed that the group effect of the cardiac cycle duration, systolic_ frequency, Diastolic_ frequency, Systolic_ intensity, Diastolic_ intensity, S1_intensity and S2_ intensity varied significantly, and there was no interaction effect. The DMS group has longer S2_duration, less heart tone frequency and less heart sound intensity than the CON group according to the main effect analysis, see Figure 1-3 *and supplementary file 3 Table1-2*.


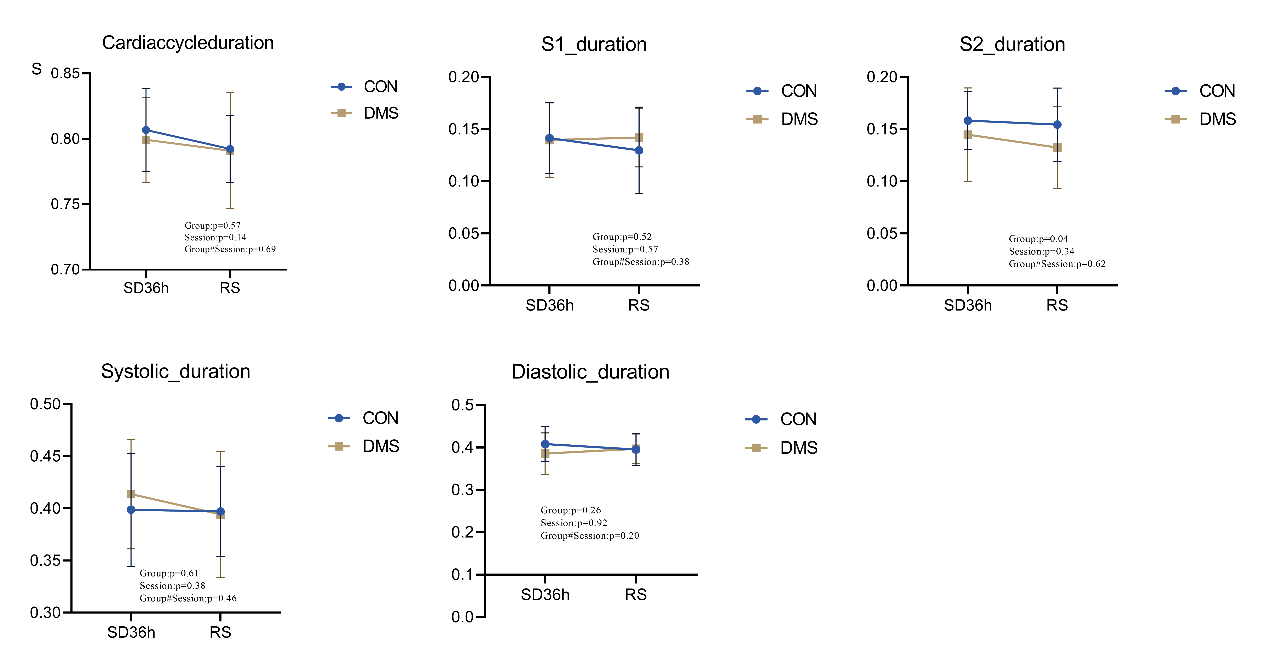


**Figure 1** Bivariate analysis of the cardiac index in the second auscultation area -Duration


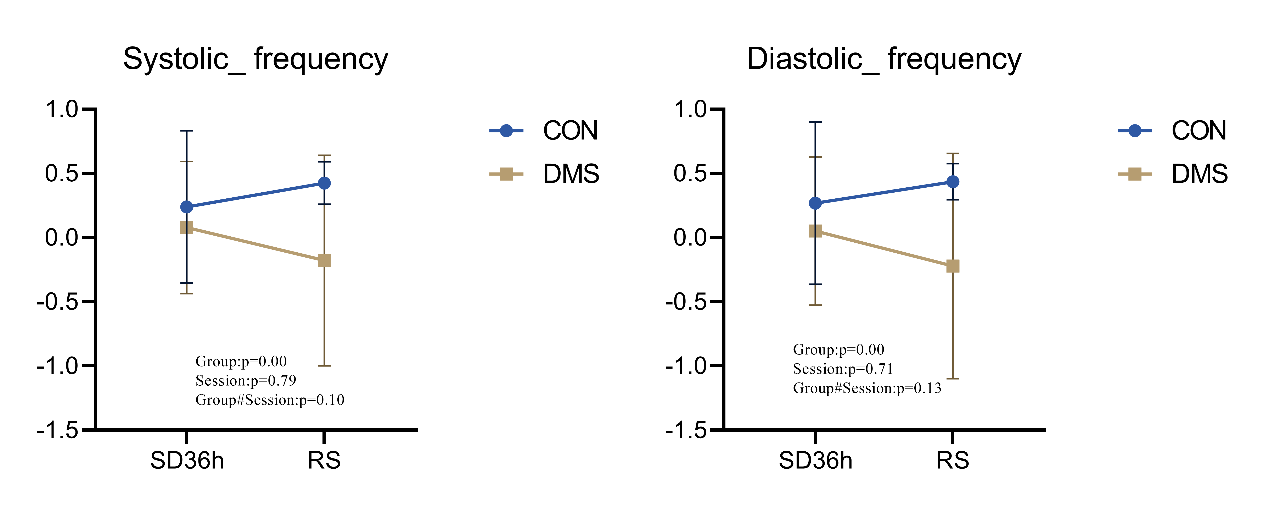


**Figure 2** Bivariate analysis of the cardiac index in the second auscultation area -Frequency


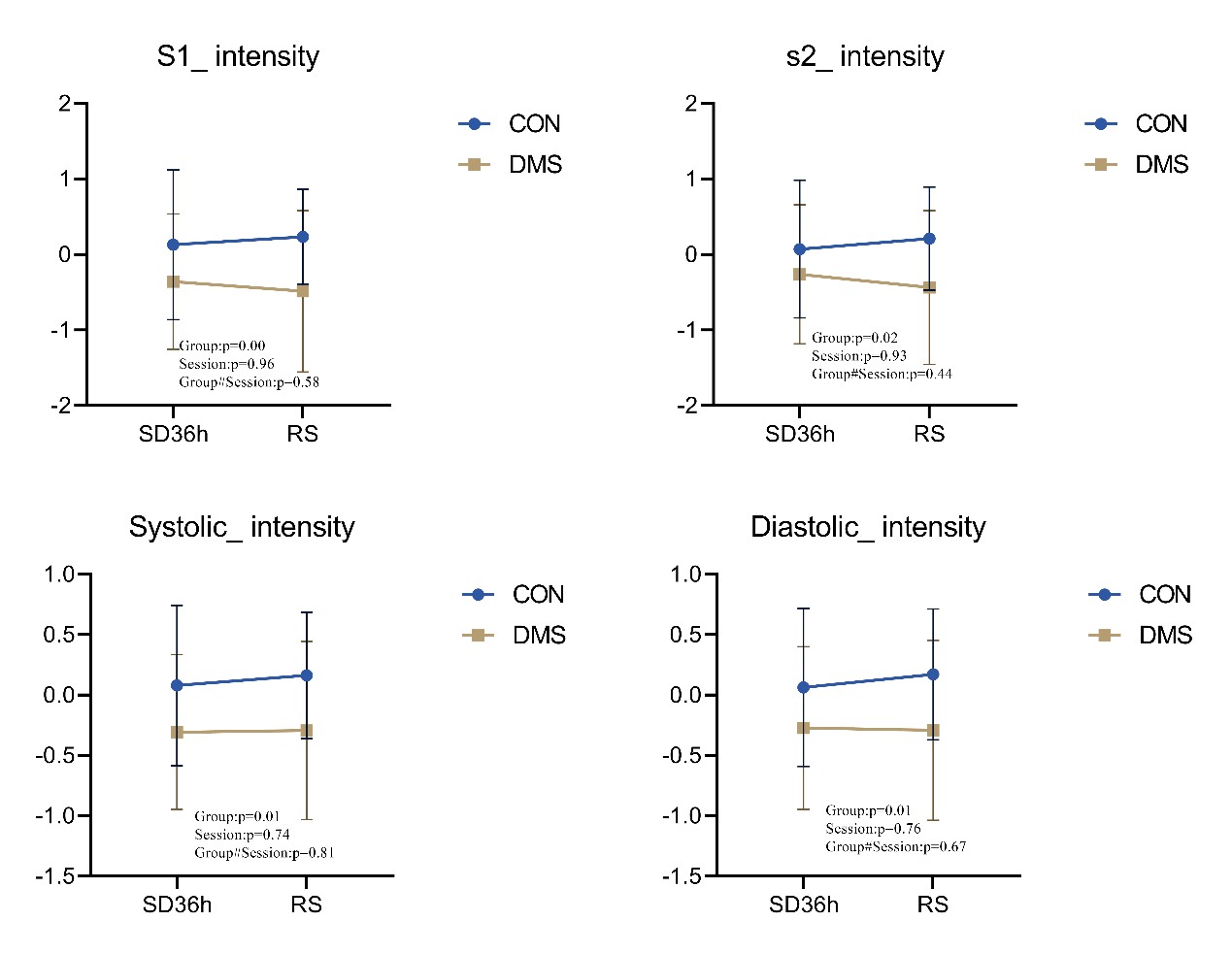


**Figure 3** Bivariate analysis of the cardiac index in the second auscultation area -Intensity

**2.The third auscultation area**

The results of the two-factor analysis disclosed that the group effect of the cardiac cycle duration, systolic_ frequency, Diastolic_ frequency, Systolic_ intensity, Diastolic_ intensity, S1_intensity and S2_ intensity varied significantly, and there was no interaction effect. The DMS group has less heart tone frequency and less heart sound intensity than the CON group according to the main effect analysis, see Figure 4-6 *and supplementary file 3 Table3-4.*


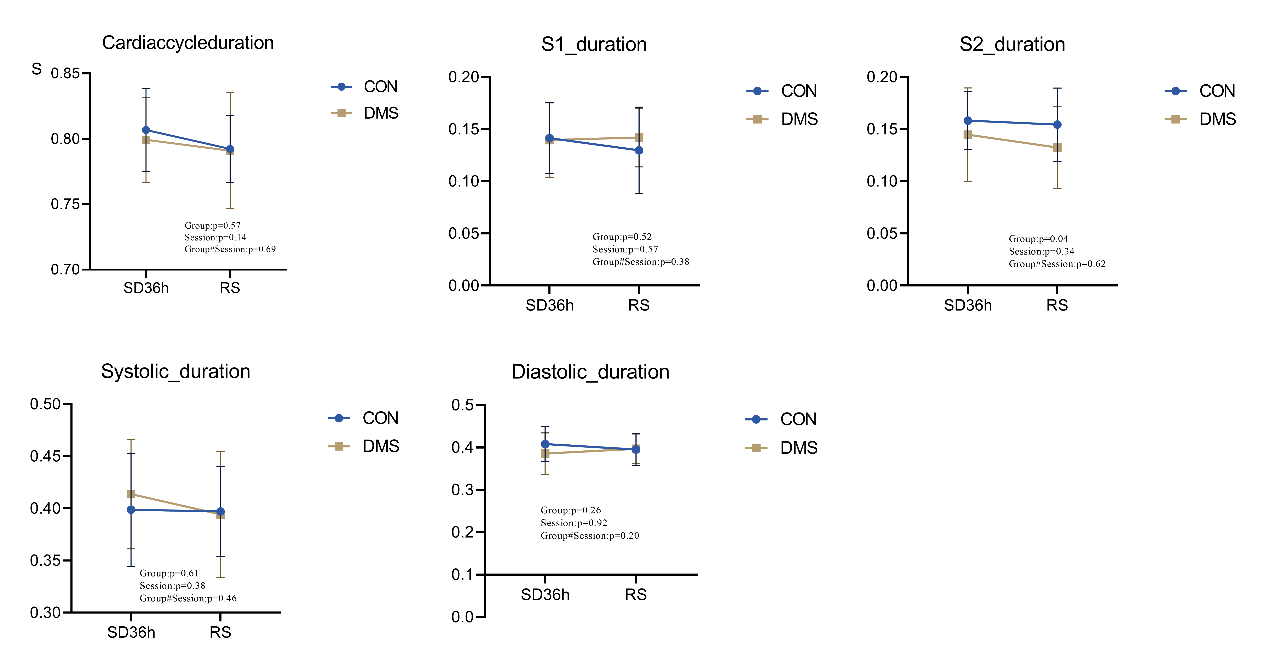


**Figure 4** Bivariate analysis of the cardiac index in the third auscultation area -Duration


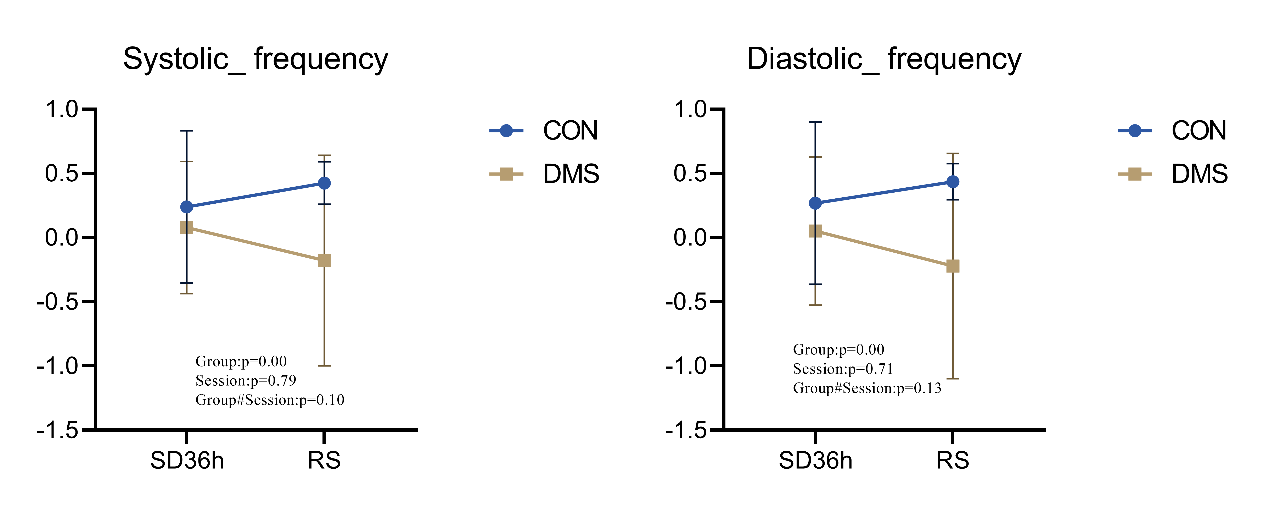
**Figure 5** Bivariate analysis of the cardiac index in the third auscultation area -Frequency


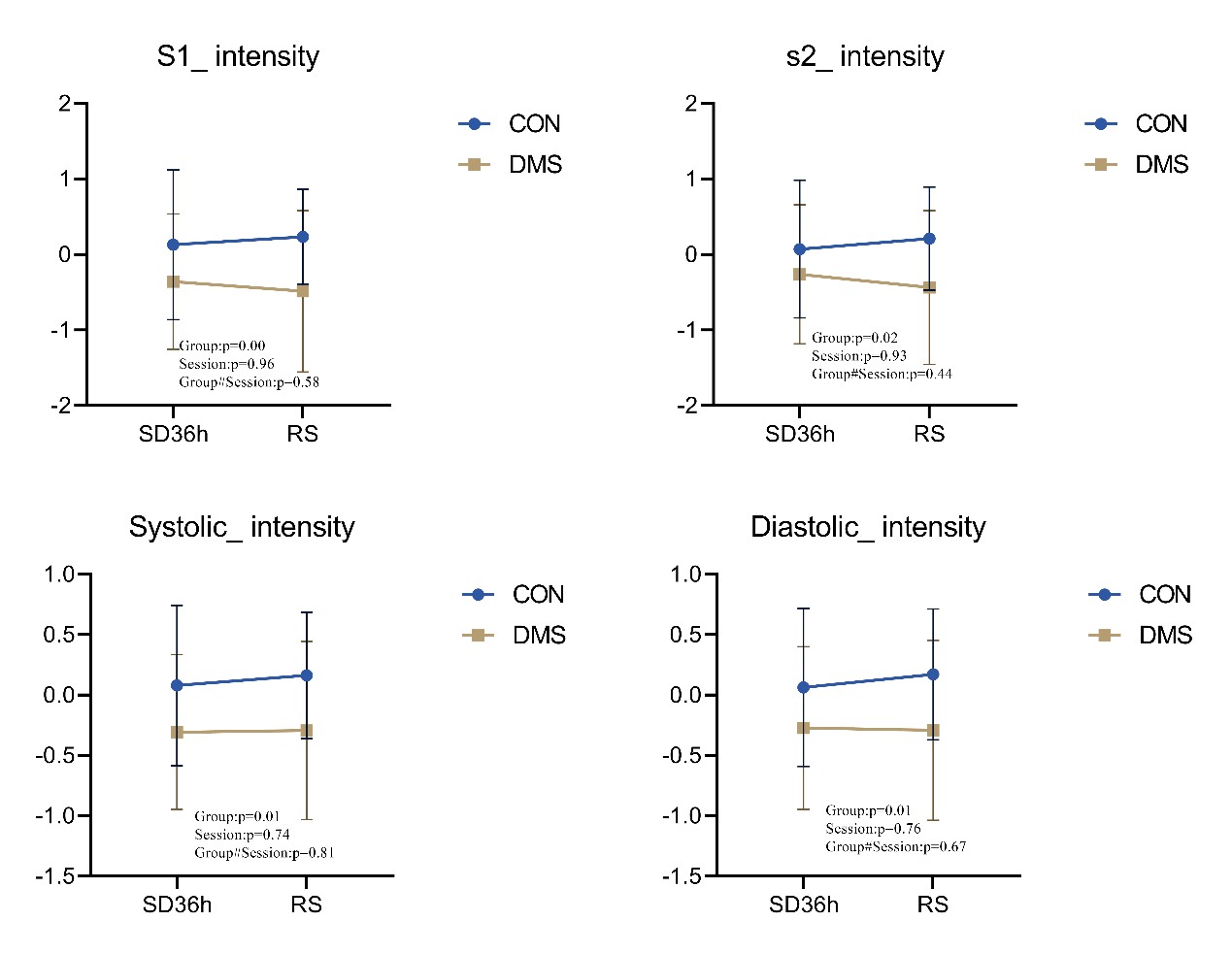


**Figure 6** Bivariate analysis of the cardiac index in the third auscultation area -Intensity

**3.The fourth auscultation area**

The results of the two-factor analysis disclosed that the group effect of the cardiac cycle duration, systolic_ frequency, Diastolic_ frequency, Systolic_ intensity, Diastolic_ intensity, S1_intensity and S2_ intensity varied significantly, and there was no interaction effect. The DMS group has less heart tone frequency and less heart sound intensity than the CON group according to the main effect analysis, see Figure 7-9 *and supplementary file 3 Table5-6.*


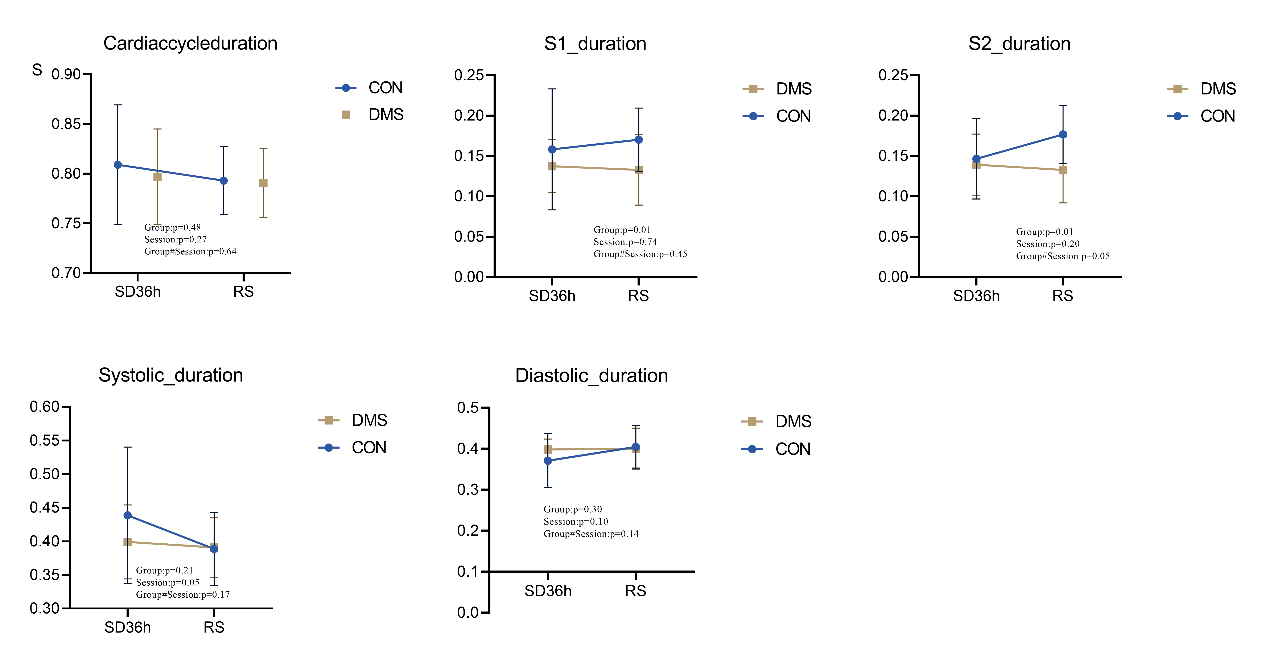


**Figure 7** Bivariate analysis of the cardiac index in the fourth auscultation area -Duration


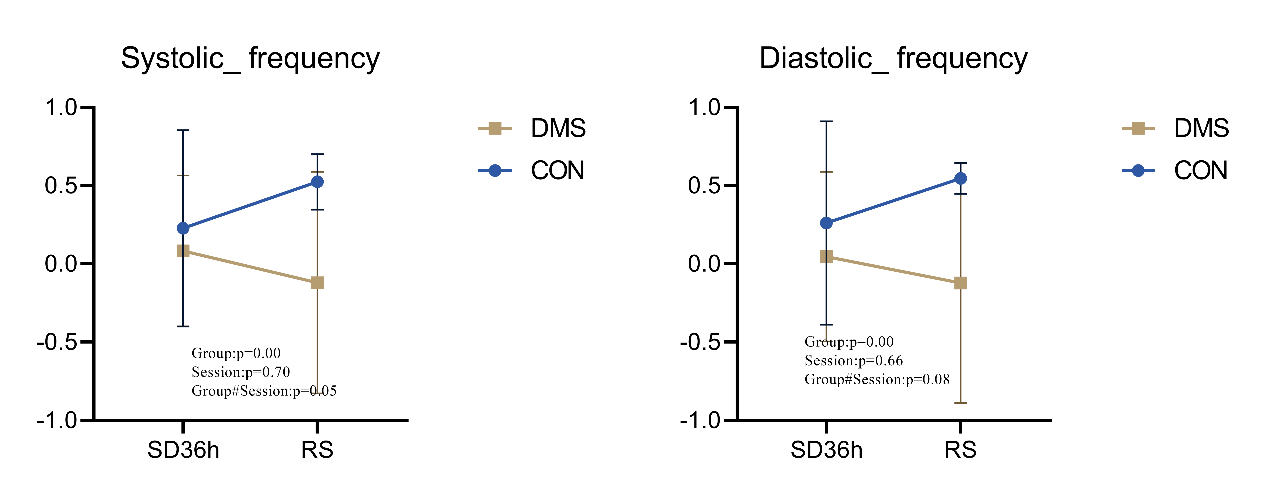


**Figure 8** Bivariate analysis of the cardiac index in the fourth auscultation area -Frequency


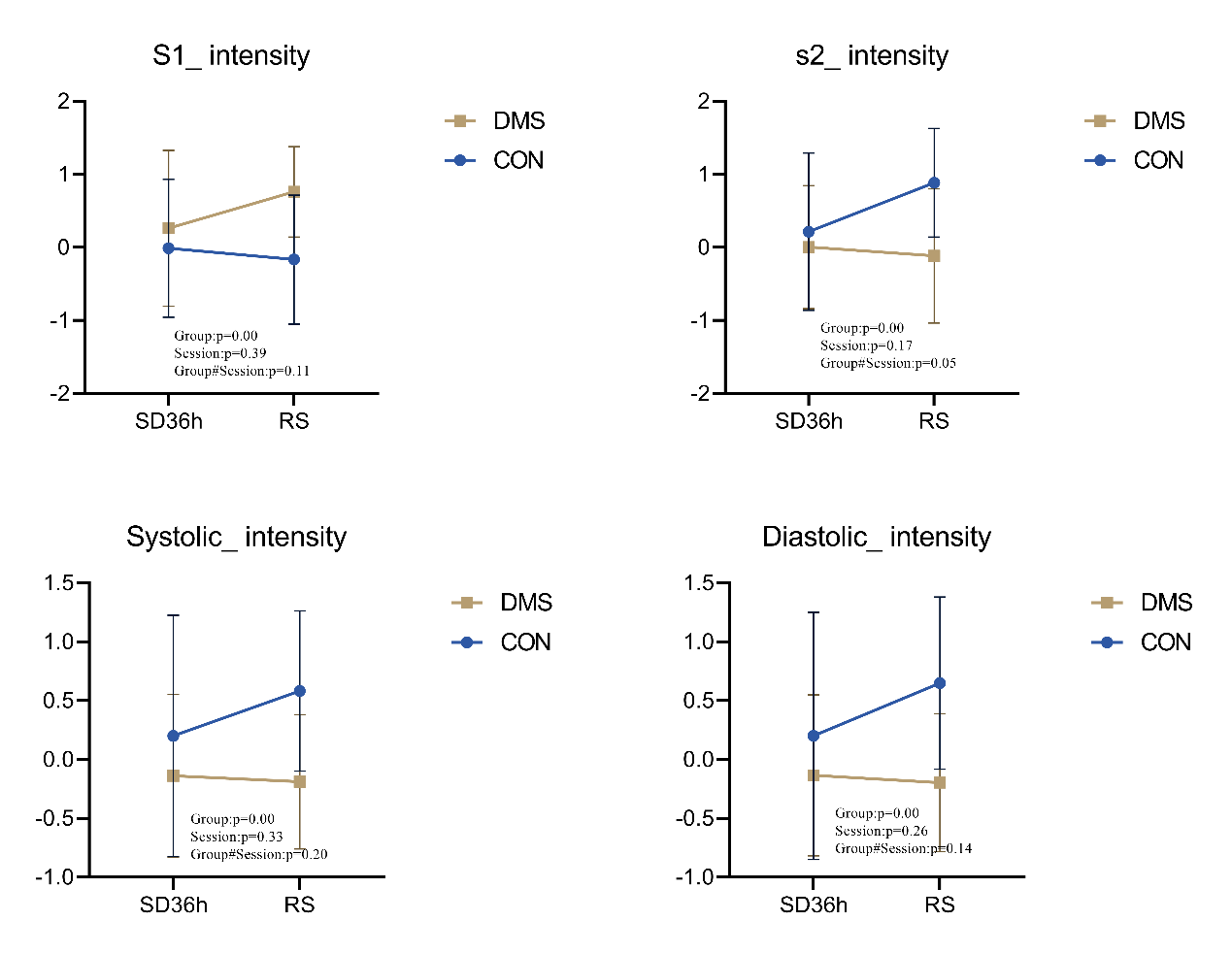


**Figure 9** Bivariate analysis of the cardiac index in the fourth auscultation area -Intensity

**4.The fifth auscultation area**

The results of the two-factor analysis disclosed that the group effect of the cardiac cycle duration, systolic_ frequency, Diastolic_ frequency, Systolic_ intensity, Diastolic_ intensity, S1_intensity and S2_ intensity varied significantly, and there was no interaction effect. The DMS group has less heart tone frequency and less heart sound intensity than the CON group according to the main effect analysis, see Figure 10-12 *and supplementary file 3 Table7-8.*


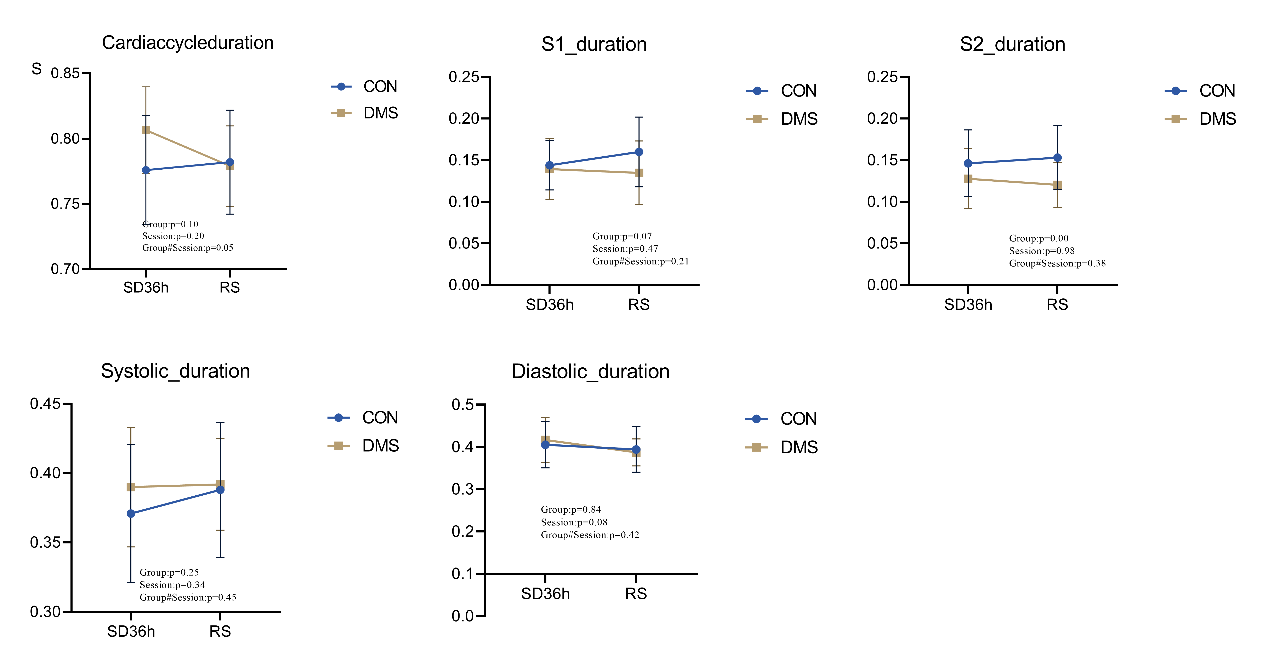
**Figure 10** Bivariate analysis of the cardiac index in the fifth auscultation area -Duration


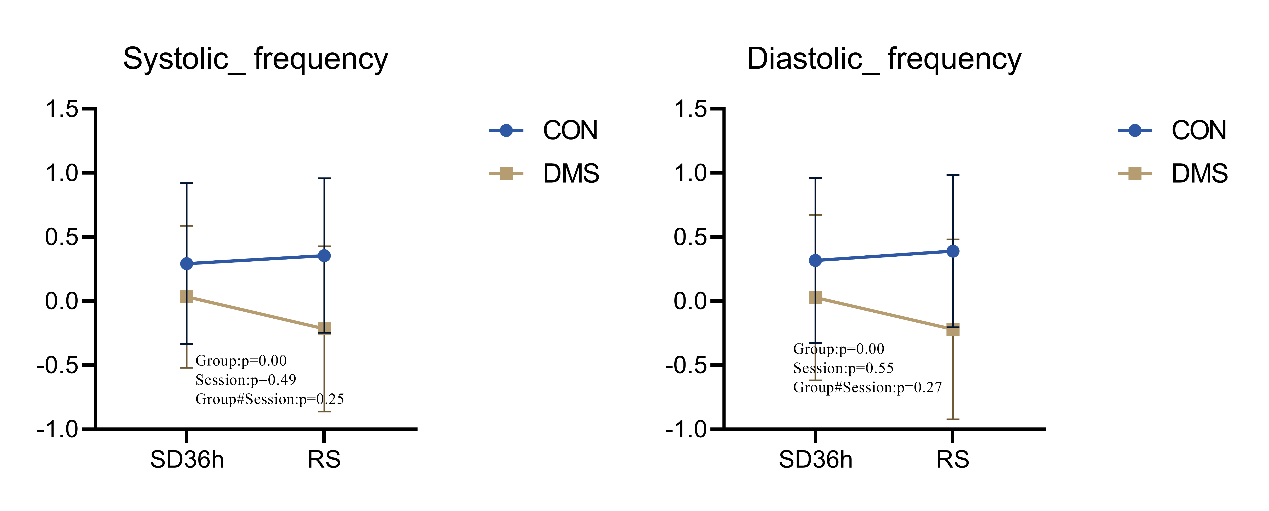


**Figure 11** Bivariate analysis of the cardiac index in the fifth auscultation area -Frequency


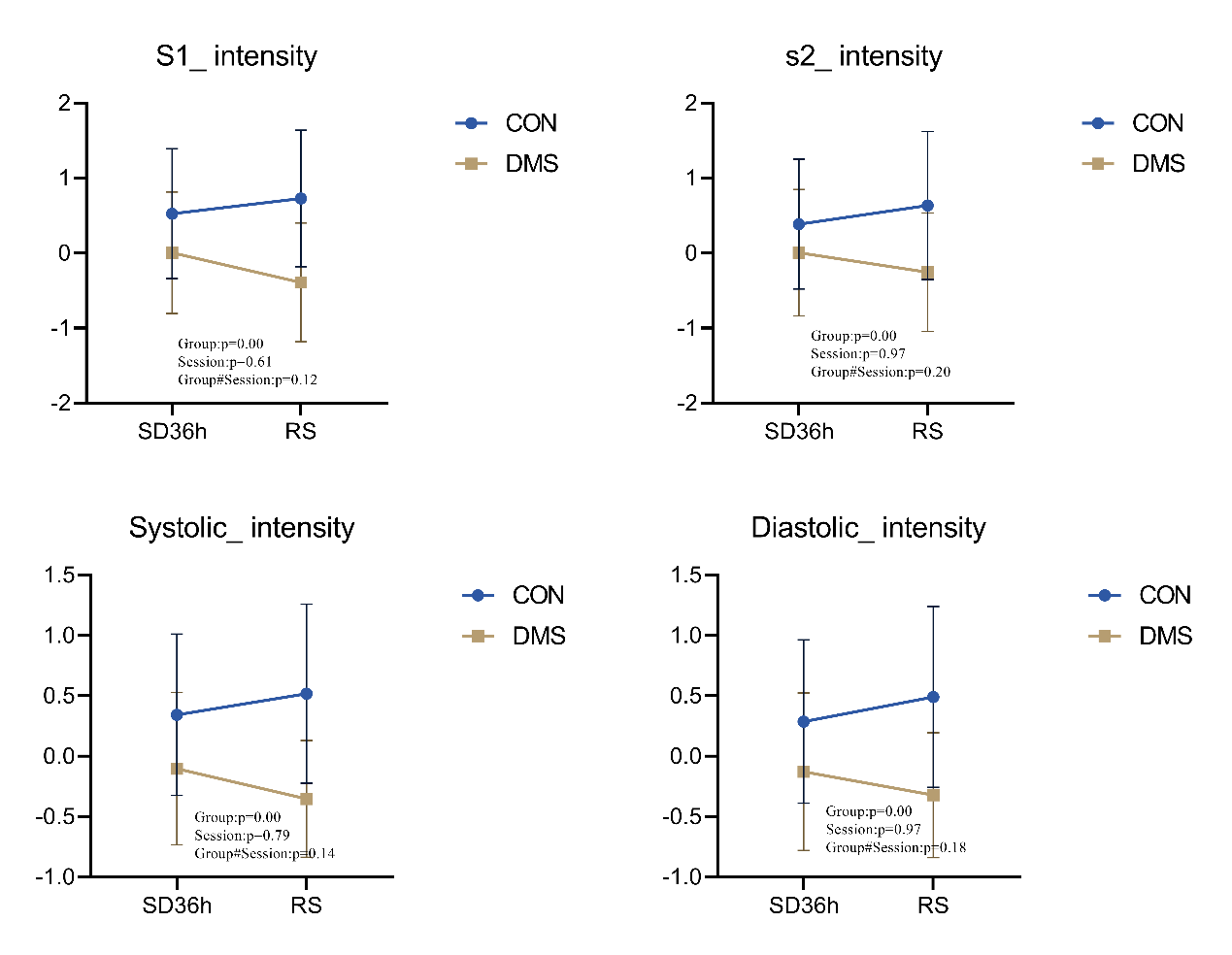


**Figure 12** Bivariate analysis of the cardiac index in the fifth auscultation area -Intensity

**Discussion**

From results of other auscultation areas, we found that the heart cycle duration in the DMS group was significant longer, while the heart sound frequency was significant decreased, the heart sound intensity was significant lower compared with the CON group, similar to those in the first auscultation area.

**Table:**

| Supplementary file 3 Table 1 Results of repeated two-factor measurements of heart sound by ANOVA in the second auscultation area | | | |
| --- | --- | --- | --- |
| Heart sound | | F | P |
| cardiaccycleduration | group | 0.33 | 0.57 |
|  | status | 2.21 | 0.14 |
|  | Session#group | 0.16 | 0.69 |
| s1_duration | group | 0.42 | 0.52 |
|  | status | 0.32 | 0.57 |
|  | Session#group | 0.78 | 0.38 |
| s2__duration | group | 4.38 | 0.04 |
|  | status | 0.92 | 0.34 |
|  | Session#group | 0.24 | 0.62 |
| systolic_duration | group | 0.26 | 0.61 |
|  | status | 0.77 | 0.38 |
|  | Session#group | 0.56 | 0.46 |
| diastolic_duration | group | 1.27 | 0.26 |
|  | status | 0.01 | 0.92 |
|  | Session#group | 1.67 | 0.20 |
| Systolic_ frequency | group | 8.50 | 0.00 |
|  | status | 0.07 | 0.79 |
|  | Session#group | 2.84 | 0.10 |
| Diastolic_ frequency | group | 9.51 | 0.00 |
|  | status | 0.14 | 0.71 |
|  | Session#group | 2.39 | 0.13 |
| Systolic_ intensity | group | 8.29 | 0.01 |
|  | status | 0.11 | 0.74 |
|  | Session#group | 0.06 | 0.81 |
| diastolic_ intensity | group | 7.18 | 0.01 |
|  | status | 0.09 | 0.76 |
|  | Session#group | 0.18 | 0.67 |
| s1_ intensity | group | 8.50 | 0.00 |
|  | status | 0.00 | 0.96 |
|  | Session#group | 0.31 | 0.58 |
| s2_ intensity | group | 5.87 | 0.02 |
|  | status | 0.01 | 0.93 |
|  | Session#group | 0.59 | 0.44 |

| Supplementary file 3 Table 2 The results of heart sound between DMS and CON group in the second auscultation area . (Mean ±SD) | | | | |
| --- | --- | --- | --- | --- |
| Heart sound | DMS |  | CON |  |
|  | SD36H | RS | SD36H | RS |
| cardiaccycleduration | 0.80±0.03 | 0.79±0.04 | 0.81±0.03 | 0.79±0.03 |
| s1_duration | 0.14±0.03 | 0.13±0.04 | 0.14±0.04 | 0.14±0.03 |
| s2__duration | 0.14±0.04 | 0.13±0.04 | 0.16±0.03 | 0.15±0.04 |
| systolic_duration | 0.41±0.05 | 0.39±0.06 | 0.40±0.05 | 0.40±0.04 |
| diastolic_duration | 0.39±0.05 | 0.40±0.03 | 0.41±0.04 | 0.40±0.04 |
| Systolic_ frequency | 0.08±0.51 | -0.18±0.82 | 0.24±0.59 | 0.42±0.17 |
| Diastolic_ frequency | 0.05±0.58 | -0.22±0.88 | 0.27±0.63 | 0.44±0.14 |
| Systolic_ intensity | -0.31±0.64 | -0.29±0.74 | 0.08±0.66 | 0.16±0.52 |
| diastolic_ intensity | -0.27±0.67 | -0.29±0.75 | 0.06±0.65 | 0.17±0.54 |
| s1_ intensity | -0.36±0.90 | -0.49±1.07 | 0.13±0.99 | 0.24±0.63 |
| s2_ intensity | -0.26±0.92 | -0.44±1.02 | 0.07±0.91 | 0.21±0.68 |

| Supplementary file 3 Table 3 Results of repeated two-factor measurements of heart sound by ANOVA in the third auscultation area | | | |
| --- | --- | --- | --- |
|  | | F | P |
| cardiaccycleduration | group | 0.15 | 0.70 |
|  | status | 0.25 | 0.62 |
|  | Session#group | 0.00 | 0.99 |
| s1_duration | group | 0.39 | 0.53 |
|  | status | 1.05 | 0.31 |
|  | Session#group | 0.23 | 0.63 |
| s2__duration | group | 0.03 | 0.85 |
|  | status | 0.10 | 0.75 |
|  | Session#group | 0.07 | 0.80 |
| systolic_duration | group | 0.50 | 0.48 |
|  | status | 0.70 | 0.41 |
|  | Session#group | 2.85 | 0.10 |
| diastolic_duration | group | 0.15 | 0.70 |
|  | status | 1.08 | 0.30 |
|  | Session#group | 2.18 | 0.14 |
| Systolic_ frequency | group | 3.82 | 0.05 |
|  | status | 0.20 | 0.66 |
|  | Session#group | 1.41 | 0.24 |
| Diastolic_ frequency | group | 4.90 | 0.03 |
|  | status | 0.00 | 0.99 |
|  | Session#group | 0.97 | 0.33 |
| Systolic_ intensity | group | 6.80 | 0.01 |
|  | status | 0.19 | 0.66 |
|  | Session#group | 0.15 | 0.70 |
| diastolic_ intensity | group | 6.42 | 0.01 |
|  | status | 0.34 | 0.56 |
|  | Session#group | 0.03 | 0.87 |
| s1_ intensity | group | 7.73 | 0.01 |
|  | status | 0.14 | 0.71 |
|  | Session#group | 0.07 | 0.80 |
| s2_ intensity | group | 5.72 | 0.02 |
|  | status | 0.68 | 0.41 |
|  | Session#group | 0.03 | 0.87 |

| Supplementary file 3 Table 4 The results of heart sound between DMS and CON group in the third auscultation area . (Mean ± SD) | | | | |
| --- | --- | --- | --- | --- |
| Heart sound | DMS |  | CON |  |
|  | SD36H | RS | SD36H | RS |
| cardiaccycleduration | 0.80±0.03 | 0.79±0.03 | 0.79±0.03 | 0.79±0.04 |
| s1_duration | 0.14±0.04 | 0.15±0.04 | 0.14±0.05 | 0.16±0.03 |
| s2__duration | 0.14±0.02 | 0.15±0.05 | 0.14±0.05 | 0.15±0.04 |
| systolic_duration | 0.41±0.04 | 0.40±0.06 | 0.39±0.04 | 0.41±0.04 |
| diastolic_duration | 0.39±0.04 | 0.39±0.06 | 0.41±0.06 | 0.38±0.06 |
| Systolic_ frequency | 0.06±0.46 | -0.04±0.77 | 0.16±0.64 | 0.38±0.52 |
| Diastolic_ frequency | 0.02±0.57 | -0.12±0.85 | 0.21±0.67 | 0.35±0.53 |
| Systolic_ intensity | -0.39±0.63 | -0.38±0.58 | -0.06±0.69 | 0.06±0.73 |
| diastolic_ intensity | -0.37±0.63 | -0.31±0.59 | -0.02±0.67 | 0.09±0.71 |
| s1_ intensity | -0.61±0.88 | -0.59±0.87 | -0.12±0.90 | 0.01±0.81 |
| s2_ intensity | -0.55±0.89 | -0.36±0.84 | -0.06±0.83 | 0.07±0.87 |

| Supplementary file 3 Table 5 Results of repeated two-factor measurements of heart sound by ANOVA in the fourth auscultation area | | | |
| --- | --- | --- | --- |
|  | | F | P |
| cardiaccycleduration | group | 0.51 | 0.48 |
|  | status | 1.22 | 0.27 |
|  | Session#group | 0.22 | 0.64 |
| s1_duration | group | 6.96 | 0.01 |
|  | status | 0.11 | 0.74 |
|  | Session#group | 0.57 | 0.45 |
| s2__duration | group | 7.84 | 0.01 |
|  | status | 1.68 | 0.20 |
|  | Session#group | 4.02 | 0.05 |
| systolic_duration | group | 1.56 | 0.21 |
|  | status | 3.82 | 0.05 |
|  | Session#group | 1.96 | 0.17 |
| diastolic_duration | group | 1.10 | 0.30 |
|  | status | 2.71 | 0.10 |
|  | Session#group | 2.21 | 0.14 |
| Systolic_ frequency | group | 10.60 | 0.00 |
|  | status | 0.15 | 0.70 |
|  | Session#group | 4.26 | 0.04 |
| Diastolic_ frequency | group | 11.68 | 0.00 |
|  | status | 0.20 | 0.66 |
|  | Session#group | 3.08 | 0.08 |
| Systolic_ intensity | group | 11.01 | 0.00 |
|  | status | 0.98 | 0.33 |
|  | Session#group | 1.66 | 0.20 |
| diastolic_ intensity | group | 11.92 | 0.00 |
|  | status | 1.28 | 0.26 |
|  | Session#group | 2.22 | 0.14 |
| s1_ intensity | group | 9.03 | 0.00 |
|  | status | 0.75 | 0.39 |
|  | Session#group | 2.66 | 0.11 |
| s2_ intensity | group | 9.24 | 0.00 |
|  | status | 1.90 | 0.17 |
|  | Session#group | 3.92 | 0.05 |

| Supplementary file 3 Table 6 The results of heart sound between DMS and CON group in the fourth auscultation area . (Mean ±SD) | | | | |
| --- | --- | --- | --- | --- |
| Heart sound | DMS |  | CON |  |
|  | SD36H | RS | SD36H | RS |
| cardiaccycleduration | 0.80±0.05 | 0.79±0.03 | 0.81±0.06 | 0.79±0.03 |
| s1_duration | 0.14±0.03 | 0.13±0.04 | 0.16±0.08 | 0.17±0.04 |
| s2__duration | 0.14±0.04 | 0.13±0.04 | 0.15±0.05 | 0.18±0.04 |
| systolic_duration | 0.40±0.06 | 0.39±0.04 | 0.44±0.10 | 0.39±0.05 |
| diastolic_duration | 0.40±0.03 | 0.40±0.05 | 0.37±0.07 | 0.40±0.05 |
| Systolic_ frequency | 0.08±0.48 | -0.12±0.71 | 0.23±0.63 | 0.52±0.18 |
| Diastolic_ frequency | 0.05±0.54 | -0.12±0.77 | 0.26±0.65 | 0.55±0.10 |
| Systolic_ intensity | -0.14±0.69 | -0.19±0.57 | 0.20±1.03 | 0.58±0.68 |
| diastolic_ intensity | -0.13±0.68 | -0.20±0.59 | 0.20±1.05 | 0.65±0.73 |
| s1_ intensity | -0.01±0.94 | -0.17±0.88 | 0.26±1.07 | 0.76±0.62 |
| s2_ intensity | 0.00±0.84 | -0.12±0.92 | 0.21±1.08 | 0.88±0.74 |

| Supplementary file 3 Table 7 Results of repeated two-factor measurements of heart sound by ANOVA in the fifth auscultation area | | | |
| --- | --- | --- | --- |
|  | | F | P |
| cardiaccycleduration | group | 2.80 | 0.10 |
|  | status | 1.69 | 0.20 |
|  | Session#group | 4.13 | 0.05 |
| s1_duration | group | 3.35 | 0.07 |
|  | status | 0.52 | 0.47 |
|  | Session#group | 1.60 | 0.21 |
| s2__duration | group | 9.94 | 0.00 |
|  | status | 0.00 | 0.98 |
|  | Session#group | 0.77 | 0.38 |
| systolic_duration | group | 1.35 | 0.25 |
|  | status | 0.91 | 0.34 |
|  | Session#group | 0.58 | 0.45 |
| diastolic_duration | group | 0.04 | 0.84 |
|  | status | 3.25 | 0.08 |
|  | Session#group | 0.66 | 0.42 |
| Systolic_ frequency | group | 9.37 | 0.00 |
|  | status | 0.48 | 0.49 |
|  | Session#group | 1.33 | 0.25 |
| Diastolic_ frequency | group | 9.62 | 0.00 |
|  | status | 0.35 | 0.55 |
|  | Session#group | 1.22 | 0.27 |
| Systolic_ intensity | group | 21.10 | 0.00 |
|  | status | 0.07 | 0.79 |
|  | Session#group | 2.20 | 0.14 |
| diastolic_ intensity | group | 17.47 | 0.00 |
|  | status | 0.00 | 0.97 |
|  | Session#group | 1.83 | 0.18 |
| s1_ intensity | group | 18.80 | 0.00 |
|  | status | 0.26 | 0.61 |
|  | Session#group | 2.49 | 0.12 |
| s2_ intensity | group | 10.56 | 0.00 |
|  | status | 0.00 | 0.97 |
|  | Session#group | 1.69 | 0.20 |

| Supplementary file 3 Table 8 The results of heart sound between DMS and CON group in the fifth auscultation area . (Mean ±SD) | | | | |
| --- | --- | --- | --- | --- |
| Heart sound | DMS |  | CON |  |
|  | SD36H | RS | SD36H | RS |
| cardiaccycleduration | 0.81±0.03 | 0.78±0.03 | 0.78±0.04 | 0.78±0.04 |
| s1_duration | 0.14±0.04 | 0.13±0.04 | 0.14±0.03 | 0.16±0.04 |
| s2__duration | 0.13±0.04 | 0.12±0.03 | 0.15±0.04 | 0.15±0.04 |
| systolic_duration | 0.39±0.04 | 0.39±0.03 | 0.37±0.05 | 0.39±0.05 |
| diastolic_duration | 0.42±0.05 | 0.39±0.03 | 0.41±0.05 | 0.39±0.05 |
| Systolic_ frequency | 0.03±0.56 | -0.22±0.64 | 0.29±0.63 | 0.36±0.60 |
| Diastolic_ frequency | 0.03±0.65 | -0.22±0.70 | 0.32±0.64 | 0.39±0.59 |
| Systolic_ intensity | -0.10±0.63 | -0.35±0.48 | 0.34±0.67 | 0.52±0.74 |
| diastolic_ intensity | -0.13±0.65 | -0.32±0.52 | 0.29±0.68 | 0.49±0.75 |
| s1_ intensity | 0.00±0.81 | -0.39±0.79 | 0.53±0.87 | 0.73±0.91 |
| s2_ intensity | 0.01±0.84 | -0.25±0.79 | 0.39±0.87 | 0.63±0.99 |
