# Supplementary material for: The effect of deep magnetic stimulation on the cardiac-brain axis post-sleep deprivation: a pilot study
Source: Front Neurosci. 2025 Jan 10;18:1464299. doi: 10.3389/fnins.2024.1464299 (PMC11757894; doi:10.3389/fnins.2024.1464299)
Supplement: Supplementary file 4 [file Data_Sheet_4.docx]

Supplementary file 4

fMRI FC data acquisition and analysis process

**1. Collection of fMRI data**

All subjects were scanned by Discovery MR750 3.0T (GE Healthcare) in University of Electronic Science and Technology of China, using echo plane imaging (EPI)-BOLD sequence, 3DT1 sequence and T2WI sequence. Scan parameters of T1 sequence structure image.TR= 1800ms, TE=2.39ms, flip angle=8°, number of slices=224, slice thickness=0.8 mm, slice gap=0.4 mm, field of view=256mm*256mm, matrix size=256*256. Scan parameters of (EPI)-BOLD sequence function image. TR=2000ms, TE=30ms, field of view=220mm*220mm, number of slices=32, slice thickness=4mm, slice gap=0.8mm, flip angle=90 °, matrix size =64*64, number of volumes=240, the scans lasted for 480s. During the scan, the subjects were asked to lie on their back in the scanner with their heads secured with a sponge. Keep your body and head still. Make their bodies as relaxed and comfortable as possible. Earplugs are used to limit the potential impact of scanner noise. Before scanning, subjects read and sign the subject agreement, and the staff explained the precautions to subjects. At the beginning of each scan, the instrument operator used a microphone to remind the participants to stay awake and not think about anything during the scan. After the scan, subjects were asked if they remained awake during the scan and were confirmed by the subjects.

**2. Preprocess of fMRI data**

Data preprocessing was based on MATLAB 2013b, and resting state BOLD data were preprocessed using Statistical Parameter Mapping software (SPM12, http://www.fil.ion.ucl.ac.uk/spm) and Data Processing & Analysis for Brain Imaging (DPABI, http://rfmri. org/dpabi) using of the Mricorn toolkit to convert the original DICOM format data to NII format data. Then, data at the first 10 time points were deleted to stabilize the subject, after which a slice timing was performed to correct the difference at each time point. The head motion parameters were estimated by translating the subject's brain in each direction and the rotation on each axis as the head motion parameters. The head motion range of all subjects' BOLD data was within the motion threshold (the translational or rotational motion parameters were less than 2.5mm or 2.5°). In the normalization step, the DARTEL registration method was used. After each functional body was registered to the standard MNI space, it was resampled into 3 * 3 * 3 (mm) cubes, and 6 * 6 * 6 (mm) Gaussian smoothing was performed to reduce the spatial noise and noise generated by standardization. Remove first-order linear trends from preprocessed data, regression covariates (including Friston 24 head motion parameters, white matter signals, cerebrospinal fluid signals, and global average signals), and low-frequency filtering (0.01-0.08Hz)^1^.

**3. Statistical analysis**

In order to study the functional connectivity patterns of DBS, voxel by voxel FC calculations were performed on the whole brain based on two seed points (Frontal-Inf-Orb-L, Insula-L). Perform within group 2 * 2 analysis of variance using SPM12 (http://www.fil.ion.ucl.ac.uk/spm). Conduct post hoc analysis using paired sample t-test. All results were corrected for multiple comparisons (p<0.001) using the Error Detection Rate (FDR) method^2^.

**4. The significance of functional connectivity**

Functional connectivity is a concept used to measure the relationship between brain regions^3^. Numerous studies have confirmed that the brain is an organic whole. When the human brain processes information, the interconnections and communications between several brain regions are active ^4^. Some studies validated that insufficient sleep may cause the synapse of dendritic spines to shrink, while SD can promote synaptic enhancement and weaken the synaptic efficacy of the hippocampus^5^. Other studies indicated that SD could reduce the central behavioral system related to attention and state stability, affecting the central nervous system and changes in brain metabolism and neural activation^6^.

**5. Results**

5.1 2 * 2 within group analysis of variance


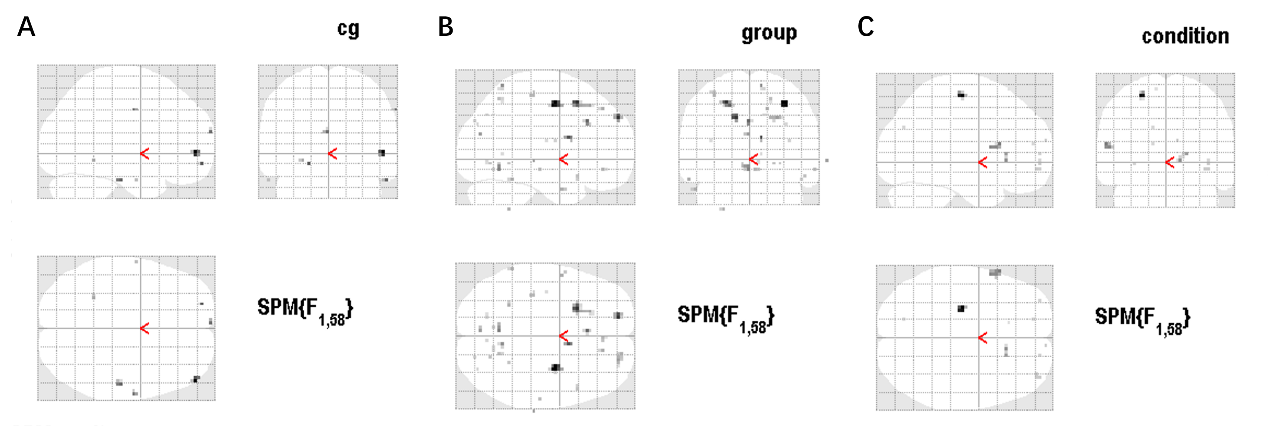


Figure 1 Frontal_ Inf_ Orb Analysis of variance results of functional connectivity in L brain area, A: interaction effect, B: intergroup effect, C: intra group effect


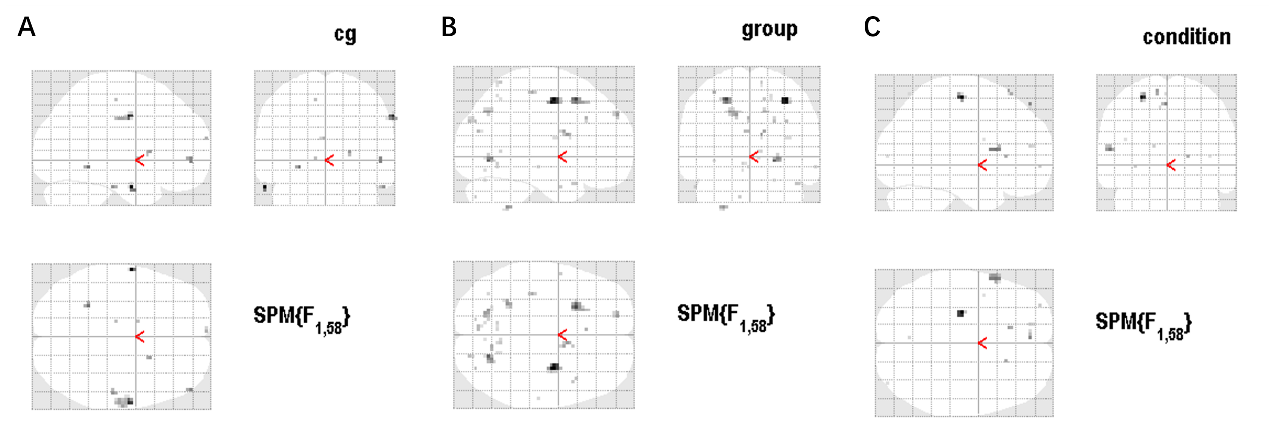


Figure 2 Insula Analysis of variance results of functional connectivity in L brain area, A: interaction effect, B: intergroup effect, C: intra group effect

5.2 Paired T Test


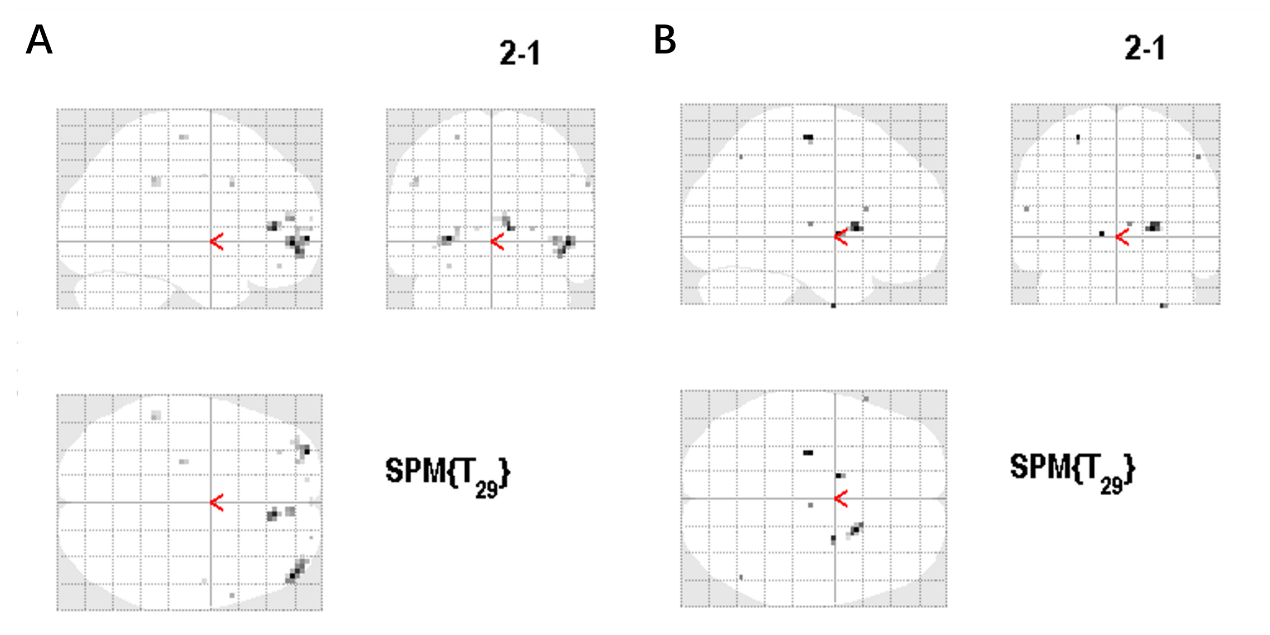


Figure 3 Frontal_ Inf_ Orb Paired T-test analysis results of functional connectivity in the L brain area, A: interaction effects, B: magnetic stimulation group


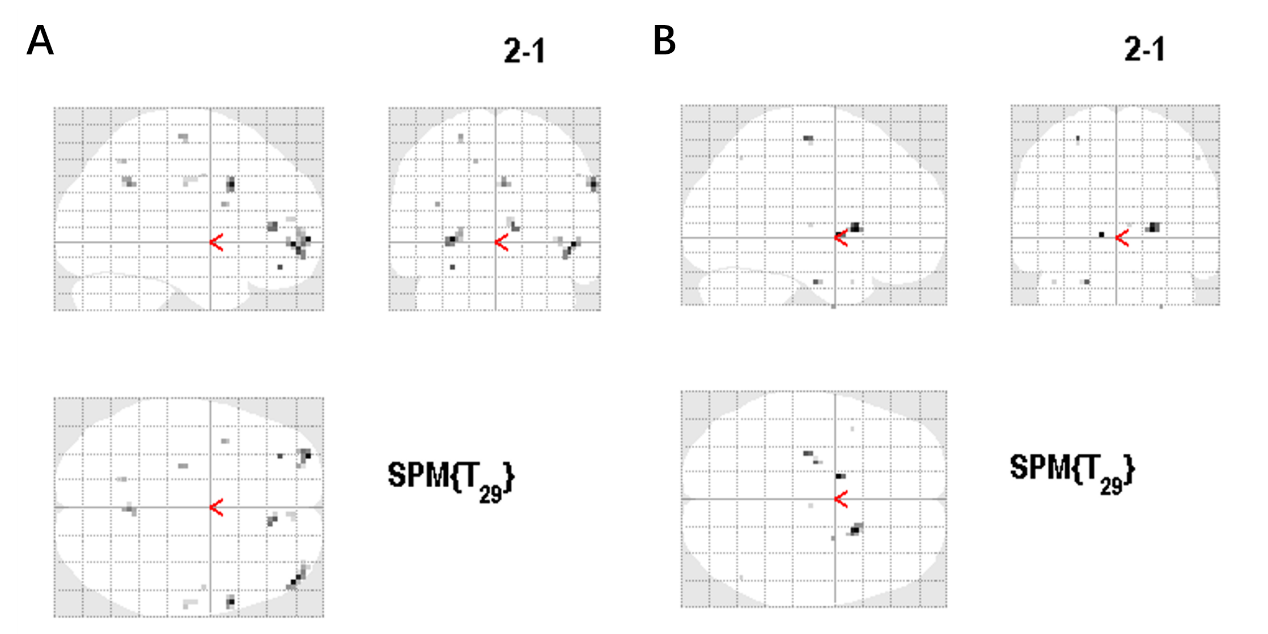


Figure 4 Insula Paired T-test analysis results of functional connectivity in the L brain area, A: interaction effects, B: magnetic stimulation group

**6. Summarize**

Figures 1 and 2 respectively show the main effects, inter group effects, and intra group effects of the functional connectivity between the (Frontal_ Inf_ Orb_L ,Insula_L) and the whole brain in the blank group and the magnetic stimulation group after sleep deprivation and sleep recovery. Moreover, no differences were found in the paired t-tests in Figures 3 and 4, which may indicate that during sleep recovery, The functional connectivity between these two brain regions and the whole brain did not change over time or the changes were not significant enough to cause significant changes, and the intervention measure: magnetic stimulation did not cause any changes in the functional connectivity of this brain region. This indicates that a brief night of restorative sleep or magnetic stimulation may not have restored the connectivity between these two brain regions and other brain regions, indicating that 36 hours of sleep deprivation may be irreversible in this regard. It may also indicate that our restorative sleep duration is too short, or that only one 20-minute magnetic stimulation cannot restore the functional connectivity between these two brain regions ^7^. We may need to increase the duration of restorative sleep or increase the duration and quantity of magnetic stimulation to further investigate the effects of intervention measures on the recovery of brain functional connectivity.

**References**

1. Pedersen M, Zalesky A. Intracranial brain stimulation modulates fMRI-based network switching. *Neurobiol Dis* **156**, 105401 (2021).

2. Worsley KJ, Marrett S, Neelin P, Vandal AC, Friston KJ, Evans AC. A unified statistical approach for determining significant signals in images of cerebral activation. *Hum Brain Mapp* **4**, 58-73 (1996).

3. Stolzberg D, Butler BE, Lomber SG. Effects of neonatal deafness on resting-state functional network connectivity. *Neuroimage* **165**, 69-82 (2018).

4. Wang C*, et al.* Large-Scale Internetwork Functional Connectivity Mediates the Relationship between Serum Triglyceride and Working Memory in Young Adulthood.

5. Raven F, Van der Zee EA, Meerlo P, Havekes R. The role of sleep in regulating structural plasticity and synaptic strength: Implications for memory and cognitive function. *Sleep Med Rev* **39**, 3-11 (2018).

6. Basner M, Rao H, Goel N, Dinges DF. Sleep deprivation and neurobehavioral dynamics. *Curr Opin Neurobiol* **23**, 854-863 (2013).

7. Sun J*, et al.* Abnormal dynamic functional connectivity after sleep deprivation from temporal variability perspective. *Hum Brain Mapp* **43**, 3824-3839 (2022).
